# Serotonergic control of valence processing in amygdalo- striaterminalis circuits

**DOI:** 10.3389/fncir.2026.1906095

**Published:** 2026-10-26

**Authors:** Ignitius Ezekiel Lim, Olalekan Michael Ogundele

**Affiliations:** Department of Comparative Biomedical Sciences, Louisiana State University School of Veterinary Medicine, Baton Rouge, LA, United States

**Keywords:** amygdala, bed nucleus of the stria terminalis (BNST), coding, dorsal raphe nucleus (DRN), affective processing, serotonin, valence

## Abstract

Valence, a hedonic value assigned to sensory stimuli that determines subsequent behaviors, is a fundamental organizing principle of affective states and emotional behaviors. Valence influences physiological processes across multiple organ systems — effects that may be mediated in part by the serotonergic (5-HT) system, which itself has widespread distribution throughout the body. The brain serotonergic system projects to the amygdala and its extended structure, the bed nucleus of the stria terminalis (BNST), regions heavily implicated in valence encoding. Despite their anatomical and functional connectivity, the amygdala and BNST express distinct 5-HT receptor subtypes, receive heterogeneous 5-HT projections, and process valence differently. The current study examines the significance of 5-HT projections in the computation of valence in the CeA and BNST. Here, multi-site *in vivo* electrophysiology was combined with anterograde chemogenetic modulation of 5-HT terminals in either the BNST or the CeA, while recording from both regions during valence presentation tasks where mice were exposed to the odorants of saline (neutral), opposite-sex urine (positive), and trimethylthiazoline (TMT, negative valence) in a counterbalanced manner. Neural responses were then compared across the neutral, appetitive, and aversive stimuli. Our results showed dorsal raphe 5-HT activation in the BNST supported valence coding in the CeA and BNST during stimulus presentation. During the post-stimulus phase, this modulation engaged only CeA-supported valence coding. Conversely, the inhibition of 5-HT terminals in the BNST supported local valence coding. Dorsal raphe 5-HT activation in the CeA enhanced valence coding in both the CeA and BNST only after stimulus offset. However, its suppression in the CeA enhanced valence coding in both the CeA and BNST during and after stimulus presentation. Taken together, our results showed that dorsal raphe serotonergic modulation creates a phasic response pattern in the CeA/BNST circuit by moderating response timing relative to stimulus valence.

## 1 Introduction

Valence is a key determinant of affective processing that shapes how a sensory experience is perceived, whether it will promote approach and consummatory behaviors (positive valence) or defense and avoidance (negative valence). Valence is not simply an inherent quality of an external stimulus, as it involves a sequence in which the brain’s limbic system encodes the stimulus and translates it into hedonic, emotional, and/or motivational reactions (Berridge and Kringelbach, 2015; Anderson and Adolphs, 2014). Several theoretical frameworks have conceptualized valence as the building block of emotional life, fundamental for categorizing stimuli and helping organisms adapt to dynamic environments (Berridge, 2019). In mammalian species, valence or affective evaluation contributes to the neural basis of consciousness (Panksepp, 2005, 2010; Paul et al., 2020; Feinberg and Mallatt, 2016). It follows that a “positive valence” is assigned to rewarding stimuli such as food or shelter, while aversive stimuli, such as punishment, are assigned a negative valence.

Furthermore, animals can associate pain relief with specific spatial locations, using this information to avoid discomfort (Davoody et al., 2011; Chen et al., 2024). When confronted with ambiguous stimuli, animals such as rodents typically default to a negative valence, approaching the situation cautiously as they continue to explore their environment (Harding et al., 2004; Nguyen et al., 2020). There are many overlapping brain regions that are conserved across different valence states in rodents and humans (Blanchard et al., 2001; Fanselow, 1994; Roelofs, 2017; Roelofs and Dayan, 2022; Zych and Gogolla, 2021). The majority of these brain regions are densely interconnected, forming functional networks essential for the emergence and regulation of valence (Malezieux et al., 2023).

The serotonergic (5-HT) system is important in valence encoding because of its extensive projections to brain regions involved in reward and fear processing (Muzerelle et al., 2016). In addition, brain 5-HT projections mediate bimodal, context-dependent modulation of the reward/aversion continuum (Nagai et al., 2020). Because of its role in brain reward/aversion and adaptive behavioral states, the 5-HT system is a primary target for anxiolytics such as the popular selective serotonin reuptake inhibitors (SSRIs). In addition, the 5-HT system in the dorsal raphe nucleus (DRN) is pertinent for sensory processing, learning, decision-making, wakefulness, impulsivity, sleep, reward, social behaviors, and mood regulation (Nakamura, 2013; Liu et al., 2014). Serotonergic innervation of the amygdala (Bocchio et al., 2016) and BNST (bed nucleus of the stria terminalis) (Hale et al., 2012) contributes to several adaptive behaviors associated with this system. The amygdala, a crucial brain structure for defense and survival, controls numerous emotional, motivational, and defensive behaviors, including reward or aversive behaviors (Kim et al., 2016), fear learning (Sengupta and Holmes, 2019), retention of fear-related memories (Orsini and Maren, 2012), and exploration (Gründemann et al., 2019). Over the last few years, the BNST, a vital part of the expanded amygdala, has gained considerable attention in fear models (Miles and Maren, 2019). Numerous studies have provided evidence confirming BNST’s participation in anxiety and anxiety-like behaviors (Walker et al., 2009; Brinkmann et al., 2018). Anterograde and retrograde tracing experiments have demonstrated projections of dorsal raphe serotonergic neurons (DRN 5-HT) to the central amygdala (CeA) (Liu et al., 2026) and BNST (Aklan et al., 2023). In addition, the CeA and BSNT have anatomically and functionally heterogeneous 5-HT projections (Ren et al., 2018) and express distinct 5-HT receptors (Hammack et al., 2009). Despite the complementary roles of the amygdala and BNST in discrete versus sustained affective states (Davis et al., 2010), how DRN 5-HT projections differentially influence CeA and BNST dynamics to modulate valence processing remains poorly defined. The current study combined concurrent *in vivo* recordings from the BNST and CeA with targeted anterograde serotonergic modulation. Our results showed that selective activation of 5-HT terminals in the CeA or BNST modulates neural response timing relative to the valence modality that is presented.

## 2 Materials and methods

### 2.1 Animals

A total of 51 adult male mice weighing 25–30 g were used for this study. Of these mice, 15 were C57BL/6 (*n* = 8 for retrograde tracing experiment and *n* = 7 for concurrent baseline electrophysiological recording from the BNST, CeA, and DRN). The remaining 36 were SERT-Cre mice (*n* = 32 for concurrent electrophysiological recording from the BNST and CeA with serotonergic modulation and *n* = 4 for optogenetic profiling of the basal electrophysiological properties of DRN 5-HT neurons; B6.129(Cg)-Slc6a4tm1(cre)Xz/J; The Jackson Laboratory). The IACUC committee of Louisiana State University approved the use of animals in this study. Animals were housed in standard cages with food/water ad libitum and alternating 12 h light/dark cycles.

### 2.2 Experimental design

Concurrent BNST and CeA recording with serotonergic modulation (*n* = 32 SERT-Cre mice): In 16 SERT-Cre mice, AAV-hSyn-DIO-hM3D(Gq)-mCherry was injected in the DRN (bilateral injection, 1 μL on each side) for chemogenetic activation of 5-HT terminals in the CeA (*n* = 8) or BNST (*n* = 8, Figure 1A). In a separate set of 16 SERT-Cre mice, AAV-hSyn-DIO-hM4D(Gi)-mCherry was injected into the DRN for chemogenetic inhibition of 5-HT terminals in the CeA (*n* = 8) or BNST (*n* = 8). Across experimental sessions, acute neural probes fitted with microfluid channels (Neuronexus, USA) were positioned in the CeA (16-channel: 1 × 16 or 32-channel: 4 × 8) and BNST (16-channel: 4 × 4 or 1 × 16) for multi-site neural recording and infusion of DREADD agonist 21 dihydrochloride (compound 21 (C21), water soluble, HB6124, Hello Bio, Bristol, UK) into the BNST or CeA. For each animal, baseline/spontaneous neural activity was recorded for 5 min. Afterward, C21 was infused into the BNST or CeA (0.1 μg/mL; 1.0 μL at 1.0 μL/min), and the injection time was time-locked to neural recording via a TTL input channel. After 5 mins following DREADD agonist infusion, animals were exposed to the valence odorant or saline for 30 s, followed by a 10-min post-stimulus phase. Mice were presented with 4% urine from age-matched females and 10% trimethylthiazoline (TMT) as the positive and negative stimulus, respectively. In each set of *n* = 8 mice, half were exposed to a saline-urine-TMT sequence, and the other half were exposed to a saline-TMT-urine sequence (Figure 1B).

**FIGURE 1:**
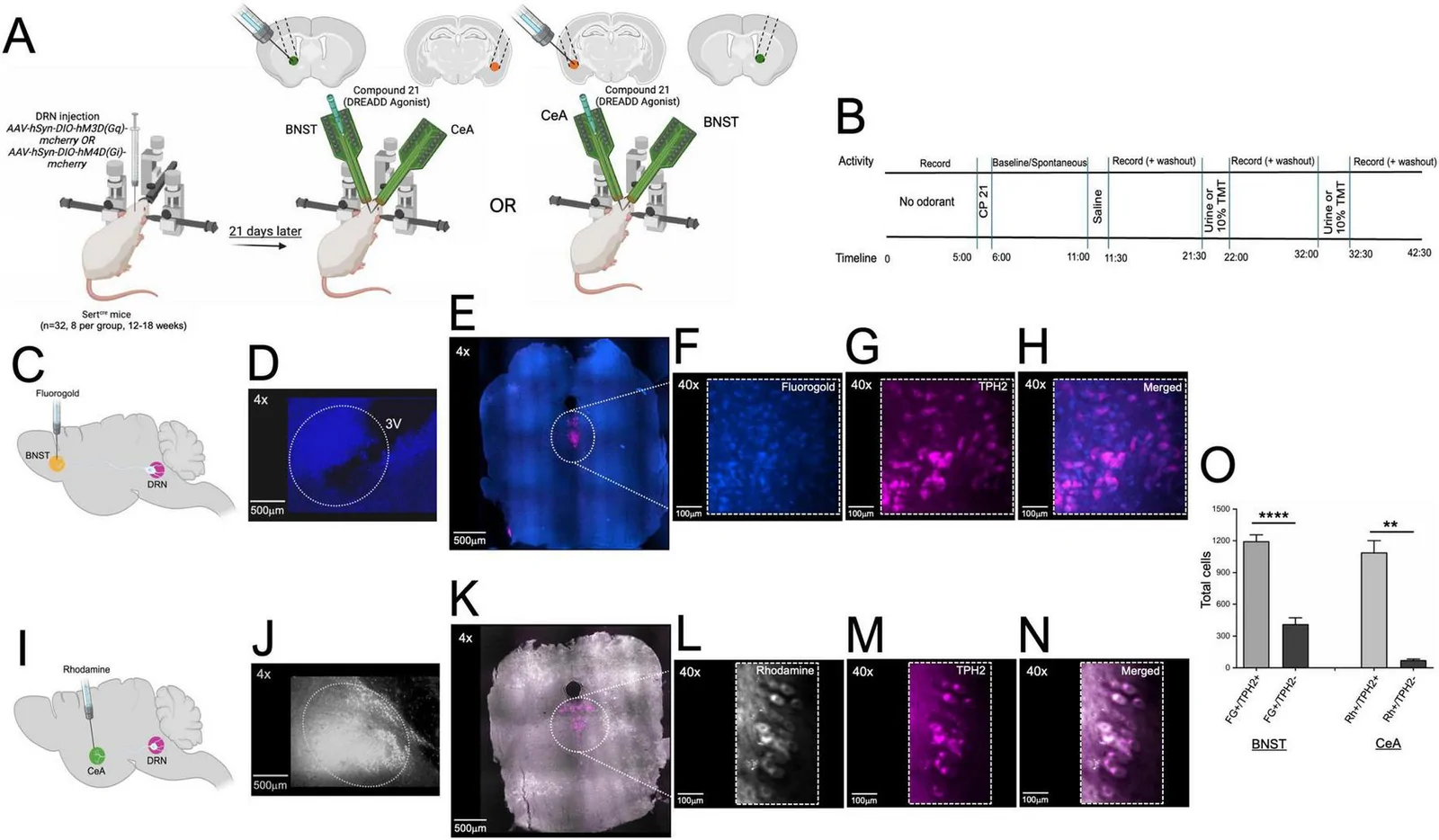
Experimental design of neural recordings and retrograde tracing of DRN 5-HT-projecting neurons in the BNST and CeA. **(A)** Schematic illustration of experimental approach. **(B)** Experimental timeline. **(C)** Schematic illustration of retrograde tracing in the BNST using adult male C57BL/6 mice (1 μL of fluorogold, *n* = 4). **(D)** Fluorogold labeling in the BNST. **(E)** Colocalization of retrogradely labeled BNST neurons with serotonergic neurons (TPH2) in the DRN at 4x magnification. **(F)** Retrogradely labeled BNST neurons in the DRN at 40x magnification. **(G)** Serotonergic neurons (TPH2) in the DRN at 40x magnification. **(H)** Merged image of retrogradely labeled BNST neurons with serotonergic neurons (TPH2) in the DRN at 40x magnification. **(I)** Schematic illustration of retrograde tracing in the CeA using adult male C57BL/6 mice (1 μL of rhodamine, *n* = 4). **(J)** Rhodamine labeling in the CeA. **(K)** Colocalization of retrogradely labeled CeA neurons with serotonergic neurons (TPH2) in the DRN at 4x magnification. **(L)** Retrogradely labeled CeA neurons in the DRN at 40x magnification. **(M)** Serotonergic neurons (TPH2) in the DRN at 40x magnification. **(N)** Merged image of retrogradely labeled CeA neurons with serotonergic neurons (TPH2) in the DRN at 40x magnification. **(O)** Comparison of *TPH2*-positive and TPH2-negative cells in the DRN following fluorogold (FG) injection in the BNST and Rhodamine (Rh) injection in the CeA (BNST: FG+/TPH2+ vs. FG+/TPH2- *p* < 0.0001, CeA: Rh+/TPH2+ vs. Rh+/TPH2- *p* = 0.0032, Paired *T*-test, **P < 0.01, ****P < 0.0001).

Retrograde tracing (*n* = 8 C57BL/6 mice): Fluorogold (100 nL, 2% w/v, Fluorochrome LLC, USA) and rhodamine (100 nL, 10% w/v, Sigma-Aldrich, USA) were injected in the BNST (*n* = 4) and CeA (*n* = 4) of separate cohorts of adult male C57BL/6 mice, respectively.

Optogenetic profiling (*n* = 4 SERT-Cre mice): AAV-hSyn-DIO-hChR2-eYFP was bilaterally injected (1 μL on each side) into the lateral parts of the dorsal raphe nucleus (DRN). Three weeks later, neural recordings were performed using an acute neural probe coupled to an optic fiber (Neuronexus, USA) positioned in the DRN (16 channel: 4 × 4). For each animal, baseline/spontaneous neural activity was recorded for 10 min. Afterward, brief pulses of blue light (470 nm; 20 Hz, 20 ms light pulse every 500 ms for 5 s) were turned on for 5 s, and activity further recorded for an additional 5 min.

Concurrent baseline BNST, CeA, and DRN recording (*n* = 7 C57BL/6 mice): Acute neural probes (Neuronexus, USA) were positioned in the BNST (16-channel: 4 × 4), CeA (16-channel: 4 × 4), and DRN (16-channel: 4 × 4) for multi-site baseline neural recording. For each animal, baseline/spontaneous neural activity was recorded for 5 min. Afterward, animals were exposed to the valence odorant or saline for 30 s, followed by a 5-min post-stimulus phase. Mice were presented with 4% urine from age-matched females as the positive valence stimulus and 10% trimethylthiazoline (TMT) as the negative stimulus. About half of the animals were exposed to a saline-urine-TMT sequence, and the other half were exposed to a saline-TMT-urine sequence.

### 2.3 Stereotaxic surgery and electrophysiology

Concurrent BNST and CeA recording with serotonergic modulation (*n* = 32 SERT-Cre mice): Mice were anesthetized with ketamine/xylazine (100:10 mg/kg), and shortly after, the plane of anesthesia was confirmed by a toe pinch test. After sedation, the animal’s head was fixed onto a stereotaxic frame, with the body on warming pads and under constant homeostatic thermoregulation. Craniotomies were performed to bilaterally expose the brain area over the dorsal raphe nucleus (DRN) at coordinates relative to the bregma (DRN, lateral: anteroposterior, AP: −4.60 mm; mediolateral, ML: ±0.4 mm; and dorsoventral, DV: 2.9 mm, Figures 1A,B). An adeno-associated viral vector (either AAV-hSyn-DIO-hM3D(Gq)-mcherry or AAV-hSyn-DIO-hM4D(Gi)-mcherry) was injected into the DRN. A total of 21 days post-surgery, mice were anesthetized with urethane/xylazine (1 g/kg: 15 mg/kg), and mounted onto a stereotaxic frame. Craniotomies were performed to expose both the BNST and CeA at coordinates relative to the bregma (BNST, lateral: AP = 0.1 mm; ML = ±0.9 mm; and CeA, lateral: AP = −1.58 mm; ML = ±2.4 mm). The dura was excised with a bent needle tip to expose the pial surface of the brain. A ground screw was positioned in the occipital bone and also connected to the ground of the recording head stage-adapter system. Two recording electrode probes fitted with microfluid channels were gently lowered into the BNST (16-channel: 4 × 4 or 1 × 16, Neuronexus, USA) and CeA (16-channel: 1 × 16 or 32-channel: 4 × 8, Neuronexus, USA) at dorsoventral coordinates relative to the pial surface (BNST: DV = 4 mm, and CeA: DV = 4.4 mm). Once the electrode probe was lowered into the BNST and CeA, and spontaneous activity was detected, the probes were kept in place for 5 min to allow the neurons to recover from physical contact with the moving electrodes. Headstages and recording probes were tethered to the amplifier controller (Intantech, USA) to monitor and record multi-unit signals, sampled at 25K/s and filtered between 0.3 and 5 KHz. Neural activity was detected in the spike view mode with analog sound outputs. The probe implant sites were verified *post hoc* by rhodamine and fluorogold labeling of the recording electrodes (Supplementary Figure 1).

Serotonergic modulation was targeted from the lateral wings of the DRN for a couple of reasons. First, the 5-HT neurons in the lateral wings exhibit greater intrinsic excitability than those in the ventral or dorsal parts (Crawford et al., 2010). Additionally, diverse 5-HT populations along the midline of the DRN (ventral, caudal, rostral, and dorsal) have been shown to colocalize with VGLUT-3 (∼80%) (Hioki et al., 2010; Liu et al., 2014), making assessment of their functional impact more difficult than in the lateral DRN, where only a very small subset is (<10%) is colocalized with VGLUT-3. Although GABA neurons are predominant in the lateral wings, only a very small minority colocalize with 5-HT (Hioki et al., 2010), making the lateral wings an ideal site for serotonergic manipulation compared to other sites. Following survival surgeries for stereotaxic injection of AAV-mediated delivery of DREADDs (designer receptors exclusively activated by designer drugs) in the DRN (Figure 1A), we performed the electrophysiological recordings under urethane-xylazine anesthesia due to its broad experimental use, sustained anesthetic depth for several hours, preservation of reflex responses, and minimal physiological disruption (Albrecht and Davidowa, 1989; Dringenberg et al., 1995; Maggi and Meli, 1986). Compared with other anesthetics, urethane is reported to maintain synaptic transmission while exerting minimal suppression of neuronal excitability (Nishikawa and MacIver, 2000; Pittson et al., 2004; Tian et al., 2012; Dyer and Rigdon, 1987; Girman et al., 1999; Ikeda and Wright, 1974; Mercer et al., 1978). Despite urethane’s advantages over many other anesthetics for physiological recordings, it is not pharmacologically inert and can therefore modulate neuronal activity to some extent (Tian et al., 2012). Nonetheless, all recordings began once the plane of anesthesia was stable, thereby minimizing any potential variability caused by anesthetic fluctuations. Furthermore, we counterbalanced the valence odorant exposure to control for confounding in the sequence of odorant presentation, including those that might be anesthesia-related.

Retrograde tracing (*n* = 8 C57BL/6 mice): Similar surgical preparation and techniques as described above were utilized here. Mice were anesthetized with ketamine/xylazine (100:10 mg/kg), and craniotomies performed to expose the BNST (for fluorogold injection in *n* = 4 animals) and CeA (for rhodamine injection in a separate cohort of *n* = 4 animals) at coordinates relative to the bregma (BNST, lateral: AP = −0.1 mm; ML = ±0.9 mm, DV = 4 mm; and CeA, lateral: AP = −1.58 mm; ML = ±2.4 mm, DV = 4.4 mm; Figures 1C–O).

Optogenetic profiling (*n* = 4 SERT-Cre mice): Similar surgical preparation and techniques to those described above were employed. Mice were anesthetized with ketamine/xylazine (100:10 mg/kg) and AAV-hSyn-DIO-hChR2-eYFP bilaterally injected (1 μ L on each side) into the lateral parts of the dorsal raphe nucleus (DRN). A total of 21 days post-injection, mice were anesthetized with urethane/xylazine (1 g/kg: 15 mg/kg). Afterward, a craniotomy was performed to expose the DRN. An acute neural probe coupled to an optic fiber (Neuronexus, USA) was positioned in the DRN (16-channel: 4 × 4) for multi-site neural recording. The probe implant sites were verified *post hoc* by rhodamine labeling of the recording electrodes (Figure 2).

**FIGURE 2:**
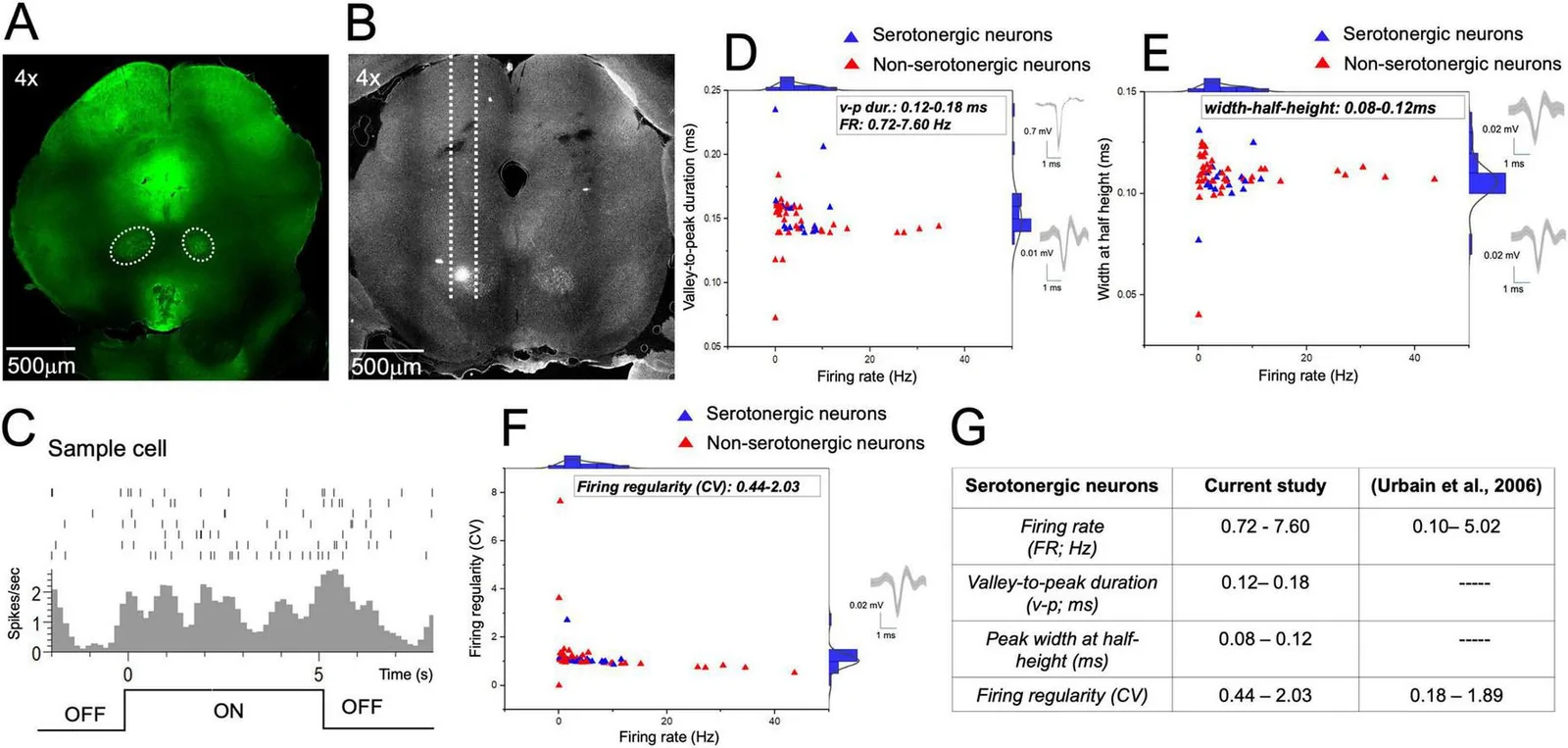
Optogenetic profiling of the basal electrophysiological properties of DRN serotonergic neurons using adult male SERT-Cre mice (*n* = 4). **(A)** Bilateral injection (1 μL on each side) of AAV-hSyn-DIO-hChR2-eYFP in the lateral parts of the dorsal raphe nucleus (DRN). **(B)** Anatomical verification of DRN recording electrode placement via rhodamine track. **(C)** A sample putative cell activated after brief pulses of blue light (470 nm; 20 Hz, 20 ms light pulse every 500 ms for 5 s) was turned on. **(D)** Marginal histogram showing the clustering of serotonergic (blue triangles; with sample waveforms) and non-serotonergic units (red triangles) sampled in the DRN by firing rate (*Hz*) and valley-to-peak duration (ms). **(E)** Marginal histogram showing the clustering of serotonergic (blue triangles; with sample waveforms) and non-serotonergic units (red triangles) sampled in the DRN by firing rate (Hz) and peak width at half-height (ms). **(F)** Marginal histogram showing the clustering of serotonergic (blue triangles; with sample waveforms) and non-serotonergic units (red triangles) sampled in the DRN by firing rate (Hz) and firing regularity (coefficient of variation; CV). **(G)** Summary table of the basal electrophysiological properties of the DRN serotonergic neurons (Urbain et al., 2006).

Concurrent baseline BNST, CeA, and DRN recording (*n* = 7 C57BL/6 mice): Similar surgical preparation and techniques as described above were used here. Mice were anesthetized with urethane/xylazine (1 g/kg: 15 mg/kg). Craniotomies were performed to expose the BNST, CeA, and DRN. Acute neural probes (Neuronexus, USA) were positioned in the BNST (16-channel: 4 × 4), CeA (16-channel: 4 × 4), and DRN (16-channel: 4 × 4) for concurrent multi-site baseline neural recording. The probe implant sites were verified *post hoc* by rhodamine and fluorogold labeling of the recording electrodes (Figure 3).

**FIGURE 3:**
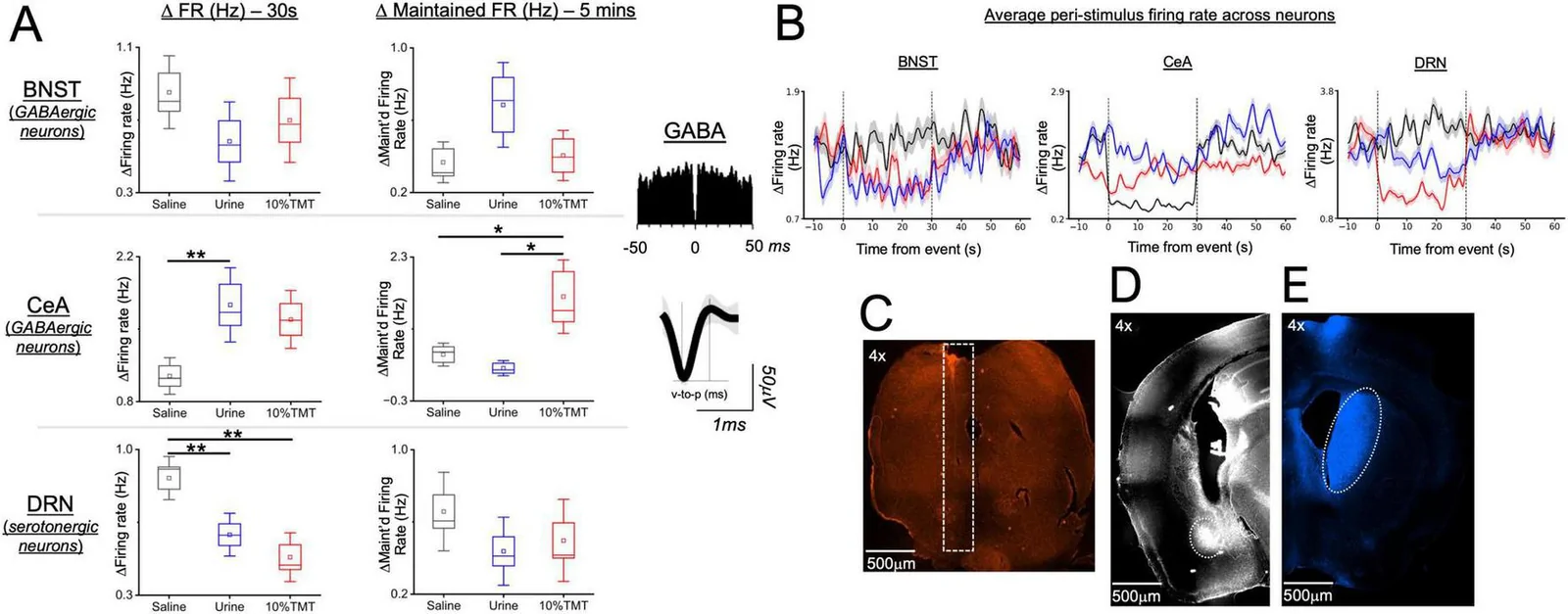
Concurrent baseline electrophysiological recording from the BNST, CeA, and DRN during odorant presentations. **(A)** Normalized firing rate and maintained firing rate in the BNST (putative GABAergic neurons), CeA (putative GABAergic neurons), and DRN (putative serotonergic neurons) during odorant presentations {[BNST, firing rate (FR): saline vs. urine *p* = 0.098; saline vs. 10% TMT *p* = 0.44; urine vs. 10% TMT *p* = 0.44; BNST, maintained FR: saline vs. urine *p* = 0.63; saline vs. 10% TMT *p* = 0.89; urine vs. 10% TMT *p* = 0.63], [CeA, firing rate (FR): saline vs. urine *p* = 0.0033; saline vs. 10% TMT *p* = 0.050; urine vs. 10% TMT *p* = 0.29; CeA, maintained FR: saline vs. urine *p* = 0.60; saline vs. 10% TMT *p* = 0.037; urine vs. 10% TMT *p* = 0.020], and [DRN, firing rate (FR): saline vs. urine *p* = 0.0055; saline vs. 10% TMT *p* = 0.0096; urine vs. 10% TMT *p* = 0.55; DRN, maintained FR: saline vs. urine *p* = 0.74; saline vs. 10% TMT *p* = 0.74; urine vs. 10% TMT *p* = 0.87]}. Additionally, a representative sample waveform and autocorrelogram of putative GABAergic units are shown in a 1 ms window and 50 ms bins, respectively. **(B)** Average perievent firing rate before, during (0–30 s; dashed lines indicate stimulus onset and offset), and after odorant presentation across neurons in the BNST, CeA, and DRN of experimental animals. **(C)** Anatomical verification of DRN recording electrode placement via rhodamine track. **(D)** Anatomical verification of CeA recording electrode placement via rhodamine track. **(E)** Anatomical verification of BNST recording electrode placement via fluorogold track (Linear mixed model with *post hoc* model-based contrasts and correction for multiple comparisons using the Holm-Bonferroni method; *P < 0.05, **P < 0.01).

### 2.4 Spike sorting and identification of putative units

Neural recordings were processed in Offline Spike Sorter (OFSS v5, Plexon USA) through principal component analysis. Interspike interval (ISI) violations were accounted for, such that putative spikes assigned to units had at least 1ms ISI. The waveforms of sorted spikes were analyzed in Neuroexplorer (Nex Technologies, USA) to determine the trough-to-peak duration (msec) of the putative units. In addition, the mean firing rate (mean FR), autocorrelogram, and ISI histogram were used in identifying serotonergic, glutamatergic, and GABAergic neurons. The identification of units was further verified by clustering in edge histograms (Supplementary Figure 2). GABAergic units from the concurrent BNST and CeA recording with serotonergic modulation had a valley-to-peak duration of less than 0.45 ms, peak width at half height of less than 0.3 ms, and variable mean FR. Due to the smaller number of sampled putative glutamatergic units from both concurrent baseline and modulation experiments, they were removed from the plots and further statistical analysis. Cells with atypical waveforms and autocorrelograms were also excluded from subsequent analysis.

### 2.5 Histology and *post hoc* verification of recording sites

After neural recording, mice were euthanized by exposure to isoflurane in a desiccator. The concentration of isoflurane was increased until loss of reflexes and cessation of respiration were observed. Cervical dislocation was then performed as a secondary physical method to ensure death. Death was confirmed by the absence of respiration, heartbeat, and toe pinch response. Transcardial perfusion was performed with phosphate-buffered saline (PBS) and 4% paraformaldehyde in PBS (4% PFA-PBS). Brains were collected, fixed in 4% PFA-PBS, and later cryopreserved in 4% PFA-PBS containing 30% sucrose. A total of 40-micron cryostat sections containing the BNST, CeA, and DRN were mounted on slides to visualize rhodamine- and fluorogold-labeled probe sites. Expression of adeno-associated viral vectors (AAVs) was confirmed in each mouse at both the DRN (injection site) and the BNST or CeA (modulation site). To visualize the colocalization of retrogradely labeled BNST or CeA neurons with 5-HT neurons in the DRN, tryptophan hydroxylase 2 (TPH2) immunofluorescence was performed on DRN sections. Sections were washed in PBS and PBS TritonX-100 (PBST). Blocking was performed in 5% normal goat serum prepared in PBST. Primary antibody incubation was performed overnight at 4°C (TPH2, catalog # PA1-778, ThermoFisher, USA). Secondary antibody labeling was performed at room temperature for 1h (Alexa Fluor 647, catalog # A-21244, ThermoFisher, USA). Fluorescent images were obtained with a Nikon NiU microscope with Moments camera (Teledyne Photometrics).

### 2.6 Statistics

Normality testing on model residuals was done using the Shapiro-Wilk test. Electrophysiological endpoints were analyzed using linear mixed-effect models (LMMs), with stimulus condition (saline, opposite-sex urine, and TMT) as a fixed effect and animal identity as a random effect to account for repeated measurements and the non-independence of multiple units recorded from the same animal. Repeated observations across stimulus conditions were modeled at the neuron level, with measurements from the same neuron treated as repeated observations and neurons nested within animals to account for within-animal dependence. *Post hoc* pairwise comparisons were performed using model-based estimated marginal means with Holm–Bonferroni correction for multiple comparisons. A summary of the linear mixed-effects model analyses is shown in Supplementary Table 1. Average line plots of the rate histogram were generated in Python (v3.13.5). Other graphs were generated using either OriginPro 2024 (OriginLab, USA) or Python. All statistical analyses were performed in Python, with statistical significance set at *p*-value < 0.05 (5%).

## 3 Results

### 3.1 Identification of putative GABAergic units

To determine how 5-HT activity in the BNST and CeA shapes valence processing, we used viral vectors to selectively manipulate 5-HT terminals in each region while simultaneously recording local circuit activity. Viral vectors expressing either an excitatory or inhibitory DREADD (hM3D(Gq) or hM4D(Gi), respectively) were injected into the DRN of SERT-Cre mice to anterogradely label 5-HT neurons. Three weeks post-surgery, neural activity was recorded simultaneously from the BNST and CeA under anesthesia, while locally modulating 5-HT terminals in either brain region (*activation of 5-HT terminals in the BNST, inhibition of 5-HT terminals in the BNST, activation of 5-HT terminals in the CeA, inhibition of 5-HT terminals in the CeA*, *n* = 8 per group) via a microfluid cannula connected to one of the neural probes (Figure 1A). After recording spontaneous activity, animals were exposed to the odorants of valence stimuli in a counterbalanced sequence of either saline-urine-TMT or saline-TMT-urine (Figure 1B). Urine from age-matched females was selected as the positive stimulus because chemosignals from opposite-sex conspecifics are innately attractive to adult male mice (Johnston and Bronson, 1982; Baum and Keverne, 2002). TMT, a synthetic constituent of fox feces, was used as the negative stimulus because it evokes robust innate defensive and avoidance responses in rodents (Takahashi et al., 2005). Concurrent neural recording from the BNST and CeA, coupled with local dorsal raphe serotonergic manipulation in either region, enables computation of valence coding within the BNST-CeA local circuits while examining how the serotonergic system influences neural dynamics during valence processing (Figures 1A,B). Viral vector expression was confirmed at the injection site in the DRN, with labeled 5-HT terminals observed in the BNST and CeA, and post-hoc histology confirmed accurate placement of recording probes in both regions (Supplementary Figure 1). Animals with off-target injections or probe misplacement were excluded from analysis. A total of BNST: *n* = 122, 56, 49, and 103, and CeA: *n* = 96, 89, 48, and 154 GABAergic units were sampled from the BNST and CeA of mice in group 1 (SERT-Cre: BNST, hM3D), group 2 (SERT-Cre: BNST, hM4D), group 3 (SERT-Cre: CeA, hM3D), and group 4 (SERT-Cre: CeA, hM4D), respectively (Supplementary Figures 2A–D). Data points in the marginal histograms correspond to individual neurons pooled across the stimulus sessions. GABAergic units had shorter action potentials (APs) with narrow waveforms, having valley-to-peak (v-p) duration around 0.45 ms or less, compared to glutamatergic cells with longer APs and wider waveforms having v-p duration greater than 0.45 ms (Supplementary Figures 2E,F).

### 3.2 Baseline valence coding in the DRN-CeA-BNST circuits

Retrograde tracing experiments performed by injecting fluorogold and rhodamine into the BNST and CeA, respectively showed colocalization of retrogradely labeled cells in the DRN with *TPH2* (Tryptophan hydroxylase 2) immunoreactivity, revealing that DRN 5-HT neurons project to the BNST (Figures 1C–H) and CeA (Figures 1I–N). In the DRN, a significant proportion of retrogradely labeled fluorogold (74.9% ± 1.9% (SEM), *p* < 0.0001) and rhodamine cells (93.9% ± 1.25% (SEM), *p* = 0.0032) in the DRN colocalized with *TPH2* (*TPH2*-positive) compared to those that did not (*TPH2*-negative; Figure 1O, Paired *T*-test). Negative *TPH2* immunoreactivity in retrogradely labeled cells from these target sites is supported by evidence from neuroanatomical tracing and functional studies showing that dorsal raphe projections to the BNST and CeA are not exclusively serotonergic – with GABAergic and glutamatergic contributions (Halberstadt and Balaban, 2006; Xu et al., 2021; Liu et al., 2014; Fortin-Houde et al., 2023; Hioki et al., 2010; Ren et al., 2018). To determine the basal electrophysiological properties of putative serotonergic neurons, a separate cohort of adult male SERT-Cre mice (*n* = 4) received bilateral injections of a viral vector expressing channelrhodopsin in the DRN to optogenetically activate and identify neurons during recordings without odorants (Figure 2). Viral expression was confirmed bilaterally in the DRN (Figure 2A), and neural probes coupled to optic fibers were verified to target the DRN (Figure 2B). Individual serotonergic neurons were identified by light-evoked responses during the stimulation period (representative cell, Figure 2C). Serotonergic neurons exhibited a valley-to-peak spike duration of 0.12–0.18 ms and firing rate of 0.72–7.60 Hz (Figure 2D), a spike width-at-half-height of 0.08–0.12 ms (Figure 2E), and firing regularity (CV) of 0.44–2.03 (Figure 2F), distinguishing them from non-serotonergic DRN neurons and consistent with previously reported values (Figure 2G; Urbain et al., 2006). These waveform and firing properties were used to classify putative serotonergic versus non-serotonergic neurons in the DRN throughout the remaining recordings in this study.

To elucidate the functionality of the DRN-CeA-BNST circuits in valence coding, concurrent baseline recording was conducted from all three brain regions using adult male C57BL/6 mice (Figure 3, *n* = 7). Results revealed a firing rate increase in the CeA during odorant presentation from urine of the opposite sex compared with the saline control (*p* = 0.0033; Figure 3A). Additionally, the maintained firing rate increased during TMT odorant presentation compared with saline (*p* = 0.037) and urine (*p* = 0.020, Figure 3A). In the DRN, the firing rate decreased during urine and TMT odorant presentations compared with the saline control (*p* = 0.0055 and *p* = 0.0096, respectively; Figure 3A). As a qualitative exploratory observation, visual inspection of the perievent firing-rate histograms qualitatively revealed distinct temporal response patterns to positive- and negative-valence stimuli in the CeA and DRN during and after stimulus presentation. In the CeA, temporal dynamics show a separable, divergent response pattern between the positive and negative stimuli during the first half (∼15 s) of the stimulus interval, converge during the second half (∼last 15 s), and diverge again following stimulus termination (Figure 3B). In the DRN, these response patterns diverge during stimulus presentation and converge starting from the last ∼5 s of the stimulus interval onwards (Figure 3B). These finer-grained temporal dynamics were not formally quantified and are not central to our conclusions, which are instead based on quantified firing-rate changes during the 30 s stimulus window and the 5 min post-stimulus period (Figure 3A). In the BNST and CeA, the activity of putative GABAergic neurons was analyzed, while that of putative serotonergic neurons was analyzed in the DRN.

Collectively, the tracing results suggest that dorsal raphe 5-HT neurons project to the BNST and CeA (Figures 1C–O). The neural dynamics in the DRN suggest it may encode valence during the exposure/presentation period (discrete phase), whereas the CeA responses suggest it may encode valence during and after exposure (sustained phase, Figure 3).

### 3.3 Valence coding following modulation of DRN 5-HT terminals in the BNST

Neural spikes were continuously sampled from the BNST and CeA during baseline and odorant presentation (Figure 4A). Perievent firing rate analysis showed an increase in firing rate following the onset of both positive and negative valence stimuli (Figure 4B). However, this increase was transient during the stimulus presentation period (0–30 s) and subsequently either decreased or returned to baseline. To visualize valence-specific neural population dynamics, principal component analysis (PCA) was applied to the neural activity vectors to reduce the high dimensionality of the neural data. For each neuron, we extracted the firing rate (FR), mean interspike interval (ISI), ISI coefficient of variation (CV), and burstiness (fraction of ISIs < 10 ms) within sliding 1-s windows across the saline control, opposite-sex urine, and TMT epochs. Following activation of DRN 5-HT terminals in the BNST, PCA revealed separable neural population patterns associated with valence (Figure 4C). Firing rate and maintained firing rate analyses were used to capture epochs during and after odorant presentation, respectively, with animal-level (left) and neuron-level (right) distributions (Figure 4D). Multi-unit recording showed that the activation of DRN 5-HT terminals in the BNST increases the firing rate (FR) of putative GABAergic units in the BNST during odorant presentations for urine of the opposite sex (positive valence stimulus, *p* < 0.0001) and 10% TMT (negative valence stimulus, *p* < 0.0001), compared to the saline control (Figure 4D). Moreover, there was a significant difference in firing rate between positive- and negative-valence stimuli, with a marked increase during TMT presentation compared to urine (*p* = 0.0012). However, the maintained firing rate showed only a marked difference between saline and TMT presentations (*p* = 0.0065; Figure 4D), with no significant difference between valence stimuli (*p* = 0.28) or between saline and urine (*p* = 0.091). Similarly, activation of 5-HT terminals in the BNST increased the firing rate of putative GABAergic units in the CeA during presentations of urine (*p* = 0.00041) and TMT (*p* < 0.0001) odorants compared with saline. In addition, the firing rate was significantly different between valence stimuli, with an increase during TMT compared to urine presentation (*p* < 0.0001). There was a significant increase in the maintained firing rate during saline (*p* < 0.0001) and TMT (*p* < 0.0001) compared to urine odorant presentation. To examine the peak dynamics, the peak firing rate and position were analyzed. For the peak firing rate, a normalization step was applied to all three stimuli (saline, urine, and TMT) by dividing each by the spontaneous (baseline) peak firing rate to account for variability in baseline recordings across sessions and to quantify stimulus-evoked responses relative to baseline activity. There were no significant differences in the normalized peak firing rate and peak position (Figure 4E) across stimuli.

**FIGURE 4:**
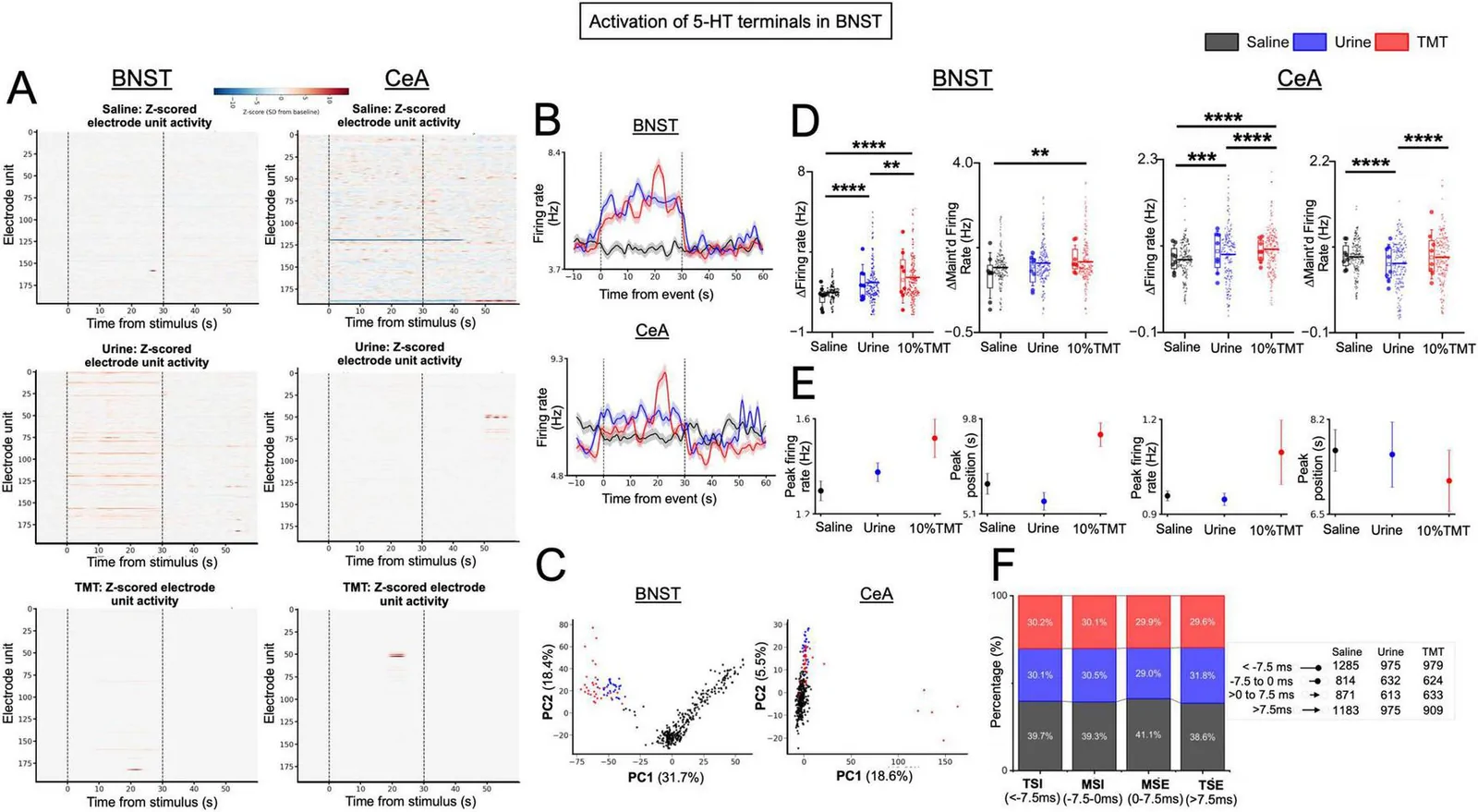
Neural coding dynamics in the BNST and CeA following activation of serotonergic (5-HT) terminals in the BNST. **(A)** Z-scored neural activity of all recorded units in the BNST and CeA before, during, and after odorant presentations. The scale is the same for all conditions. **(B)** Average perievent firing rate before, during (0–30 s; dashed lines indicate stimulus onset and offset), and after odorant presentation across all putative GABAergic neurons in the BNST and CeA of experimental animals. **(C)** Principal component analysis (PCA) of the firing rate of putative GABAergic units in the BNST and CeA following odorant presentations. **(D)** Firing rate and maintained firing rate of putative GABAergic units in the BNST and CeA following odorant presentations. Left: Animal-level distribution of the mean responses of putative GABAergic neurons from each animal (one point per animal). Right: Neuron-level distribution of individual GABAergic neuronal responses (one point per neuron). Animal identity was included as a random effect in the Linear mixed-effects model. {[BNST, firing rate (FR): saline vs. urine *p* < 0.0001; saline vs. 10% TMT *p* < 0.0001; urine vs. 10% TMT *p* = 0.0012; BNST, maintained FR: saline vs. urine *p* = 0.091; saline vs. 10% TMT *p* = 0.0065; urine vs. 10% TMT *p* = 0.28] and [CeA, FR: saline vs. urine *p* = 0.00041; saline vs. 10% TMT *p* < 0.0001; urine vs. 10% TMT *p* < 0.0001; CeA, maintained FR: saline vs. urine *p* < 0.0001; saline vs. 10% TMT *p* = 0.57; urine vs. 10% TMT: *p* < 0.0001]}. **(E)** Normalized peak firing rate and peak position in the BNST and CeA during odorant presentations (Peak FR, BNST: saline vs. urine *p* = 0.99; saline vs. 10% TMT *p* = 0.78; urine vs. 10% TMT *p* = 0.85; Peak FR, CeA: saline vs. urine *p* = 0.71; saline vs. 10% TMT *p* = 0.71; urine vs. 10% TMT *p* = 0.99, Peak position, BNST: saline vs. urine *p* = 0.73; saline vs. 10% TMT *p* = 0.45; urine vs. 10% TMT *p* = 0.90; Peak position, CeA: saline vs. urine *p* = 0.19; saline vs. 10% TMT *p* = 0.52; urine vs. 10% TMT *p* = 0.78). **(F)** Cross-correlogram analysis (CCG) based on the firing rate patterns between the BNST and CeA during odorant presentations. (Linear mixed model with post-hoc model-based contrasts and correction for multiple comparisons using the Holm-Bonferroni method; **P < 0.01, ***P < 0.001, ****P < 0.0001).

Cross-correlational histograms (CCH) were generated during CCG analysis to evaluate putative GABA-GABA connectivity between the BNST and CeA during saline, urine of the opposite sex, and TMT intervals. To ensure a high degree of anatomical connection by proximity, CCG analysis was limited to putative units on the same electrode shank with an inter-electrode distance of not more than 50 mm. Results from CCH analyses do not specify definite synaptic connections but rather describe functional coupling or connectivity between putative pairs (Lim et al., 2026). For each group, a total number of possible connections was derived by setting the neurons from the modulated brain region as the “reference” (e.g., *if BNST*) and those from the other brain region as a “target” (e.g., *CeA*). Afterward, the number of viable pairs or connections was quantified as those with interaction peaks exceeding the 99% confidence interval. Putative pairs with no viable connections (i.e., below the 99% confidence interval) were excluded from subsequent analysis. Peaks occurring at or less than 7.5 ms were considered monosynaptic, while those beyond 7.5 ms were considered transsynaptic. Positive peaks indicate excitatory connections, while negative ones indicate inhibitory connections. For all four serotonergic modulation groups, the number of connections that are either transsynaptic inhibitory (<−7.5 ms), monosynaptic inhibitory (−7.5 to 0 ms), monosynaptic excitatory (0–7.5 ms), or transsynaptic excitatory (>7.5 ms) across saline, urine, and TMT intervals was indicated (Figure 4F). Descriptive summaries of four possible functional connection types (monosynaptic excitation, transsynaptic excitation, monosynaptic inhibition, and transsynaptic inhibition) are presented without formal statistical inference. CCH analysis identifies putative functional connectivity consistent with possible synaptic interactions; however, it does not resolve the underlying synaptic mechanism, including whether it is mediated by single or co-released neurotransmitters, or by indirect network contributions. The CCG showed no changes in connectivity between the BNST and CeA across stimuli following activation of DRN 5-HT terminals in the BNST. This is consistent with the firing dynamics, which showed similar profiles in the BNST and CeA during stimulus presentation. Taken together, these results suggest that chemogenetic activation of DRN 5-HT terminals in the BNST may contribute to CeA-mediated valence coding while influencing temporal dynamics in the BNST. This cross-regional effect is consistent with the extensive reciprocal anatomical connectivity between the BNST and CeA, two core, densely interconnected nodes of the extended amygdala that share overlapping inputs and outputs and are thought to coordinate complementary aspects of threat and valence processing — the CeA mediating responses to discrete, phasic stimuli and the BNST mediating responses to sustained, diffuse threat (Davis et al., 2010). However, the CeA may influence responses to sustained fear through projections to the BNST (Davis et al., 2010). Given this direct anatomical linkage, local modulation of 5-HT terminals within the BNST may influence CeA activity indirectly via BNST output projections to the CeA, rather than reflecting a direct action of 5-HT within the CeA itself.

Similarly, following inhibition of DRN 5-HT terminals in the BNST, perievent firing increased during stimulus presentation of both positive and negative stimuli in the BNST, with decreasing post-stimulus responses (Figures 5A,B). Moreover, in the CeA, during the first 30 post-stimulus seconds, the firing appeared to be attenuated for positive and negative stimuli compared to the neutral stimulus. PCA showed distinct valence-associated population patterns (Figure 5C). Multi-unit activity showed that the inhibition of 5-HT terminals in the BNST increases the firing rate of putative GABAergic units in the BNST and CeA during odorant presentations for urine (BNST: *p* < 0.0001 and CeA: *p* < 0.0001; Figure 5D) and TMT (BNST: *p* < 0.0001 and CeA: *p* < 0.0001), compared to saline. However, there was a significant difference between valence stimuli only in the BNST, with a substantial increase in FR during TMT presentation compared to urine (*p* < 0.0001). Similarly, the maintained firing rate in the BNST and CeA increased after urine (BNST: *p* < 0.0001 and CeA: *p* < 0.0001) and TMT (BNST: *p* = 0.016 and CeA: *p* < 0.0001) odor presentations, compared to saline. Again, there was a notable difference between valence stimuli only in the BNST, with a significant reduction in maintained FR after TMT compared to urine (*p* = 0.00076). Similarly, peak firing rate and position did not change across stimuli (Figure 5E). Following inhibition of 5-HT terminals in the BNST, CCG analysis revealed varying connectivity patterns between positive and negative stimulus intervals, with a decrease in monosynaptic inhibitory connections during urine presentation (Figure 5F). Collectively, these results suggest that inhibition of DRN 5-HT terminals in the BNST may facilitate valence coding locally within the BNST only, in contrast to the cross-regional CeA effect observed with activation of the same terminals — an asymmetry that may reflect differential recruitment of the reciprocal BNST-CeA pathway depending on the direction of 5-HT modulation.

**FIGURE 5:**
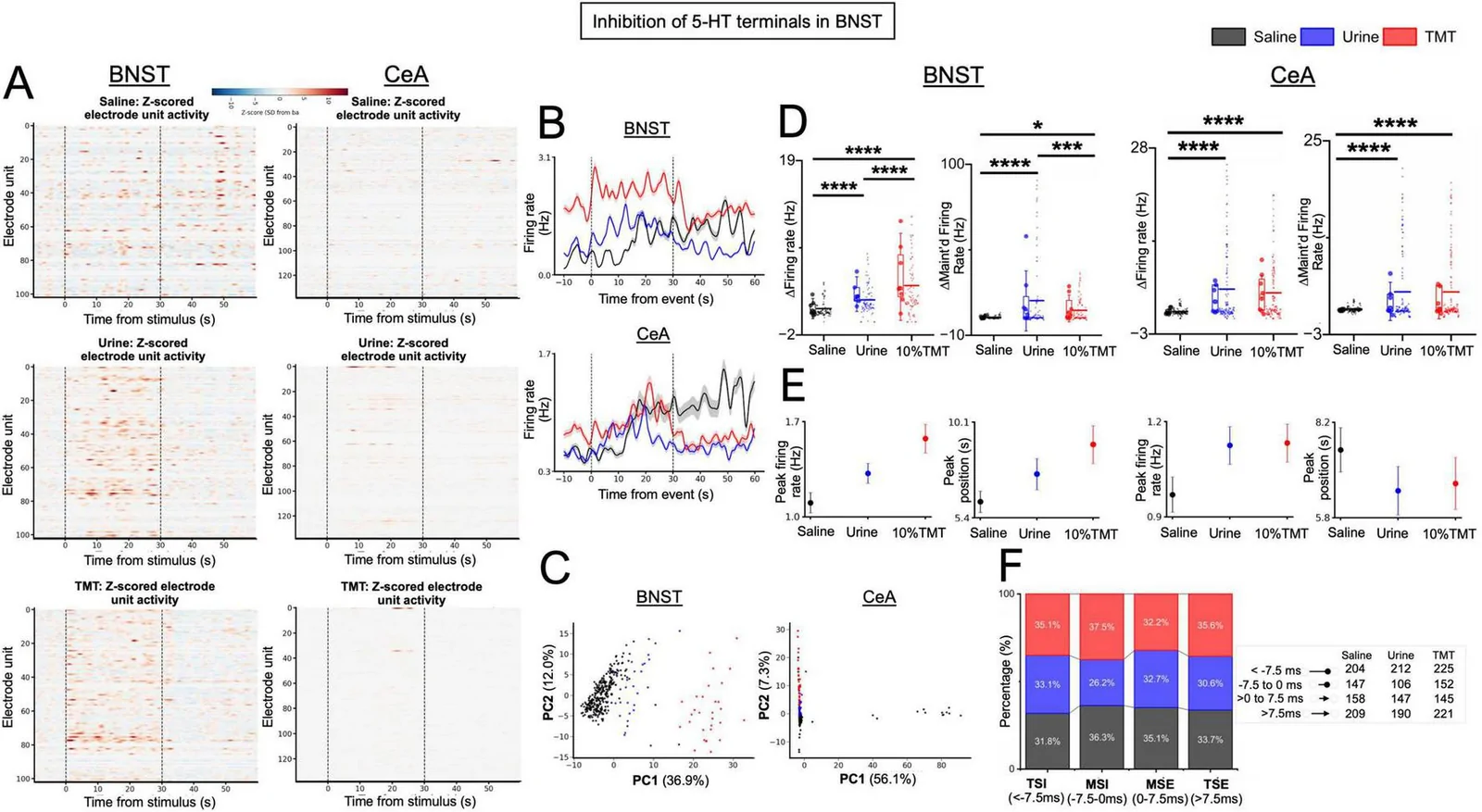
Neural coding dynamics in the BNST and CeA following inhibition of serotonergic (5-HT) terminals in the BNST. **(A)** Z-scored neural activity of all recorded units in the BNST and CeA before, during, and after odorant presentations. The scale is the same for all conditions. **(B)** Average perievent firing rate before, during (0–30 s; dashed lines indicate stimulus onset and offset), and after odorant presentation across all putative GABAergic neurons in the BNST and CeA of experimental animals. **(C)** Principal component analysis (PCA) of the firing rate of putative GABAergic units in the BNST and CeA following odorant presentations. **(D)** Firing rate and maintained firing rate of putative GABAergic units in the BNST and CeA following odorant presentations. Left: Animal-level distribution of the mean responses of putative GABAergic neurons from each animal (one point per animal). Right: Neuron-level distribution of individual GABAergic neuronal responses (one point per neuron). Animal identity was included as a random effect in the Linear mixed-effects model. {[BNST, firing rate (FR): saline vs. urine *p* < 0.0001; saline vs. 10% TMT *p* < 0.0001; urine vs. 10% TMT *p* < 0.0001; BNST, maintained FR: saline vs. urine *p* < 0.0001; saline vs. 10% TMT *p* = 0.016; urine vs. 10% TMT *p* = 0.00076] and [CeA, FR: saline vs. urine *p* < 0.0001; saline vs. 10% TMT *p* < 0.0001; urine vs. 10% TMT *p* = 0.17; CeA, maintained FR: saline vs. urine *p* < 0.0001; saline vs. 10% TMT *p* < 0.0001; urine vs. 10% TMT *p* = 0.43]}. **(E)** Normalized peak firing rate and peak position in the BNST and CeA during odorant presentations (Peak FR, BNST: saline vs. urine *p* = 0.46; saline vs. 10% TMT *p* = 0.99; urine vs. 10% TMT *p* = 0.46; Peak FR, CeA: saline vs. urine *p* = 0.90; saline vs. 10% TMT *p* = 0.37; urine vs. 10% TMT *p* = 0.18, Peak position, BNST: saline vs. urine *p* = 0.99; saline vs. 10% TMT *p* = 0.89; urine vs. 10% TMT *p* = 0.96; Peak position, CeA: saline vs. urine *p* = 0.93; saline vs. 10% TMT *p* = 0.76; urine vs. 10% TMT *p* = 0.54). **(F)** Cross-correlogram analysis (CCG) based on the firing rate patterns between the BNST and CeA during odorant presentations. (Linear mixed model with *post hoc* model-based contrasts and correction for multiple comparisons using the Holm-Bonferroni method; *P < 0.05, ***P < 0.001, ****P < 0.0001).

### 3.4 Valence coding following modulation of DRN 5-HT terminals in the CeA

Similarly, neural spikes were continuously sampled from the BNST and CeA during baseline and odorant presentation (Figure 6A). In both regions, perievent firing increased during stimulus presentation and subsequently decreased or returned to baseline (Figure 6B). Following activation of DRN 5-HT terminals in the CeA, population activity patterns associated with positive and negative valence remained separable from saline (neutral) but were not clearly distinctive from each other (Figure 6C). Moreover, multi-unit recording showed that the activation of DRN 5-HT terminals in the CeA increases the firing rate (FR) of putative GABAergic units in the BNST and CeA during odorant presentations for urine (CeA: *p* < 0.0001 and BNST: *p* < 0.0001) and TMT (CeA: *p* < 0.0001 and BNST: *p* < 0.0001), compared to the saline control (Figure 6D). However, there were no significant differences in the FR of the BNST and CeA between urine and TMT presentations (CeA: *p* = 0.15 and BNST: *p* = 0.68). In contrast, the maintained FR, which captures encoding after stimulus presentations, increased in the CeA and BNST after TMT presentation compared to saline (CeA: *p* = 0.0063 and BNST: *p* < 0.0001) and urine (CeA: *p* = 0.0060 and BNST: *p* < 0.0001). Moreover, there was a robust increase in maintained FR of the BNST after urine presentation compared to saline (*p* = 0.0068). Across stimuli, the peak firing rate and position remained consistent, with no differences (Figure 6E). Consistent with the firing response during stimulus presentation, CCG analysis revealed similar connectivity between the BNST and CeA across stimuli following activation of DRN 5-HT terminals in the CeA (Figure 6F). Activation of DRN 5-HT terminals in the CeA disrupted valence discrimination in both the CeA and BNST during stimulus presentation, but this disruption did not persist after stimulus offset. Given the dense reciprocal connectivity reported between the CeA and BNST, one possible explanation for this cross-regional effect is that 5-HT-driven changes in CeA output propagate to the BNST via their shared reciprocal projections, rather than reflecting an independent action of 5-HT within the BNST itself; however, we did not directly test this pathway, and an independent local effect in the BNST cannot be ruled out. The recovery of valence processing after stimulus offset suggests that this 5-HT-driven disruption is time-limited and does not permanently alter the underlying CeA-BNST circuitry, but instead transiently interferes with valence discrimination while the odorant stimulus is present.

**FIGURE 6:**
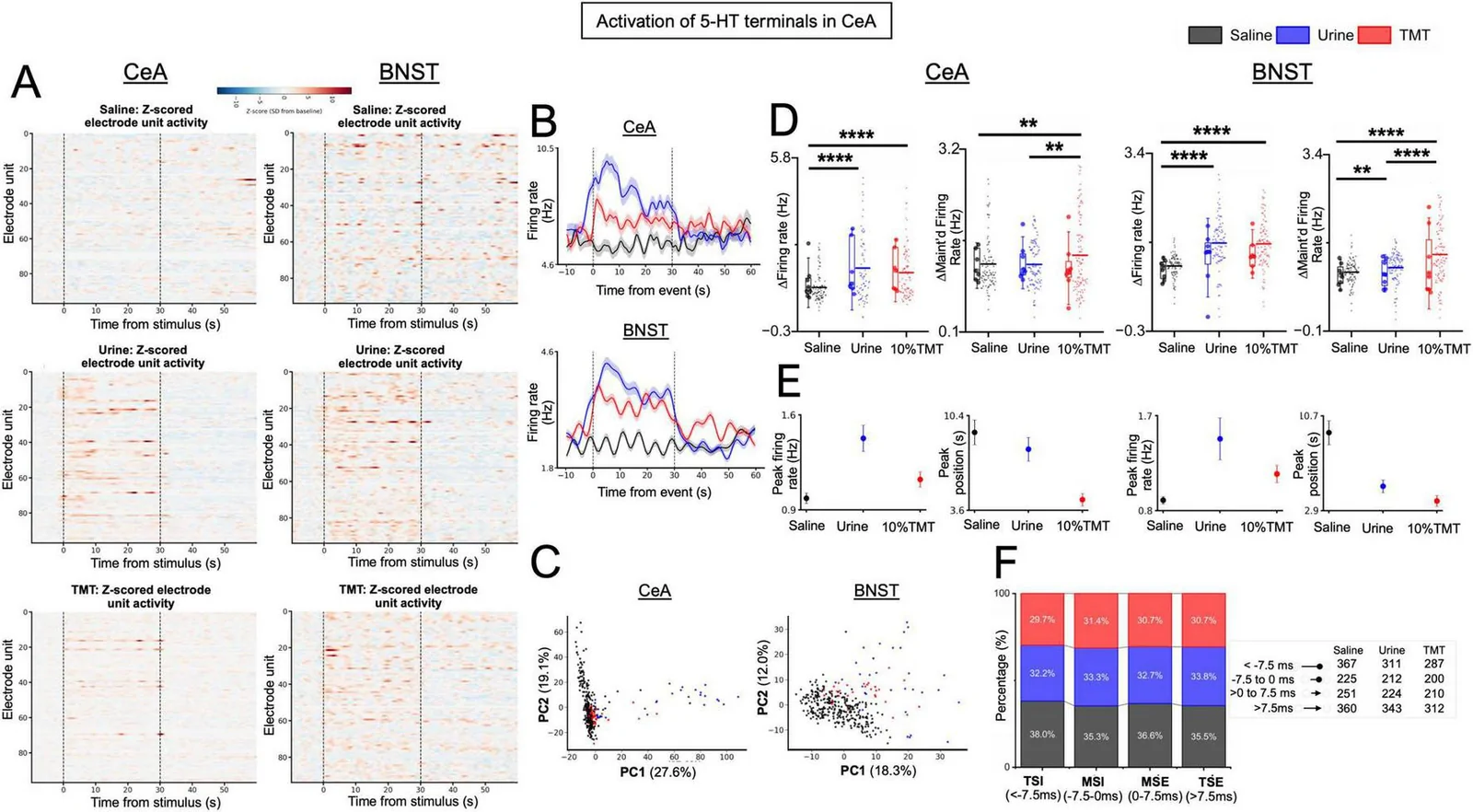
Neural coding dynamics in the BNST and CeA following activation of serotonergic (5–HT) terminals in the CeA. **(A)** Z-scored neural activity of all recorded units in the BNST and CeA before, during, and after odorant presentations. The scale is the same for all conditions. **(B)** Average perievent firing rate before, during (0–30 s; dashed lines indicate stimulus onset and offset), and after odorant presentation across all putative GABAergic neurons in the BNST and CeA of experimental animals. **(C)** Principal component analysis (PCA) of the firing rate of putative GABAergic units in the BNST and CeA following odorant presentations. **(D)** Firing rate and maintained firing rate of putative GABAergic units in the BNST and CeA following odorant presentations. Left: Animal-level distribution of the mean responses of putative GABAergic neurons from each animal (one point per animal). Right: Neuron-level distribution of individual GABAergic neuronal responses (one point per neuron). Animal identity was included as a random effect in the Linear mixed-effects model. [CeA, firing rate (FR): saline vs. urine *p* < 0.0001; saline vs. 10% TMT *p* < 0.0001; urine vs. 10% TMT *p* = 0.15; CeA, maintained FR: saline vs. urine *p* = 0.98; saline vs. 10% TMT: *p* = 0.0063; urine vs. 10% TMT: *p* = 0.0060] and [BNST, FR: saline vs. urine *p* < 0.0001; saline vs. 10% TMT *p* < 0.0001; urine vs. 10% TMT *p* = 0.68; BNST, maintained FR: saline vs. urine *p* = 0.0068; saline vs. 10% TMT *p* < 0.0001; urine vs. 10% TMT: *p* < 0.0001)). **(E)** Normalized peak firing rate and peak position in the BNST and CeA during odorant presentations (Peak FR, BNST: saline vs. urine *p* = 0.21; saline vs. 10% TMT *p* = 0.62; urine vs. 10% TMT *p* = 0.72; Peak FR, CeA: saline vs. urine *p* = 0.85; saline vs. 10% TMT *p* = 0.93; urine vs. 10% TMT *p* = 0.98, Peak position, BNST: saline vs. urine *p* = 0.64; saline vs. 10% TMT *p* = 0.99; urine vs. 10% TMT *p* = 0.66; Peak position, CeA: saline vs. urine *p* = 0.77; saline vs. 10% TMT *p* = 0.37; urine vs. 10% TMT *p* = 0.78). **(F)** Cross-correlogram analysis (CCG) based on the firing rate patterns between the BNST and CeA during odorant presentations. (Linear mixed model with post-hoc model-based contrasts and correction for multiple comparisons using the Holm-Bonferroni method; **P < 0.01, ****P < 0.0001).

Perievent firing in the BNST and CeA following inhibition of DRN 5-HT terminals in the CeA increased for positive- and negative-valence stimuli relative to the neutral during the stimulus interval. However, following stimulus termination, perievent firing of the negative stimulus remained consistent with minimal fluctuations, while that of the positive stimulus decreased (Figures 7A,B). Here, PCA showed separable valence-associated population patterns (Figure 7C). Multi-unit activity showed that the inhibition of 5-HT terminals in the CeA increases the firing rate of putative GABAergic units in the CeA and BNST during odorant presentations for urine (CeA: *p* = 0.0027 and BNST: *p* = 0.00015) and TMT (CeA: *p* < 0.0001 and BNST: *p* < 0.0001), compared to saline control (Figure 7D). There were robust FR differences in both the CeA (*p* < 0.0001) and BNST (*p* < 0.0001) between the positive and negative valence stimuli. Similarly, the maintained FR in the CeA and BNST increased after urine (CeA: *p* = 0.020 and BNST: *p* = 0.013) and TMT (CeA: *p* < 0.0001 and BNST: *p* < 0.0001) odor presentations, compared with the saline control. Again, there were marked differences in both the CeA and BNST between valence stimuli, with substantial increases in maintained FR after TMT compared to urine (CeA: *p* < 0.0001 and BNST: *p* < 0.0001). Similarly, there were no changes in peak firing and position across stimuli (Figure 7E). Across modulation groups, the majority of connections across all intervals were transsynaptic (both excitatory and inhibitory), with values above 25% for each or above 50% combined. This was expected as BNST-CeA connectivity is gated and shaped by several microcircuits, most of which are local inhibitory relays and disinhibitory motifs, often manifesting as transsynaptic coupling rather than monosynaptic. Consistent with the stimulus neural response, CCG analysis showed similar connectivity between the BNST and CeA across stimuli following inhibition of DRN 5-HT terminals in the CeA (Figure 7F). In summary, these results suggest that the inhibition of DRN 5-HT terminals in the CeA may be crucial for processing valence in both the CeA and BNST. Together, these bidirectional effects — discrimination lost with activation, preserved with inhibition — raise the possibility that CeA-projecting 5-HT input actively interferes with, rather than supports, valence discrimination in the CeA-BNST circuit, at least under these stimulus conditions. This interpretation is consistent with the CeA and BNST functioning as reciprocally interconnected, densely coupled nodes of the extended amygdala, where 5-HT-driven changes in CeA activity could plausibly propagate to the BNST via their shared reciprocal projections; however, we did not directly assess CeA-BNST connectivity or signal propagation in this study, and this remains a possibility for future investigation. Further electrophysiological analyses about the interspike interval (ISI), firing regularity (ISI coefficient of variation – CV), burst profile, and PCA are provided (Supplementary Figures 3–10).

**FIGURE 7:**
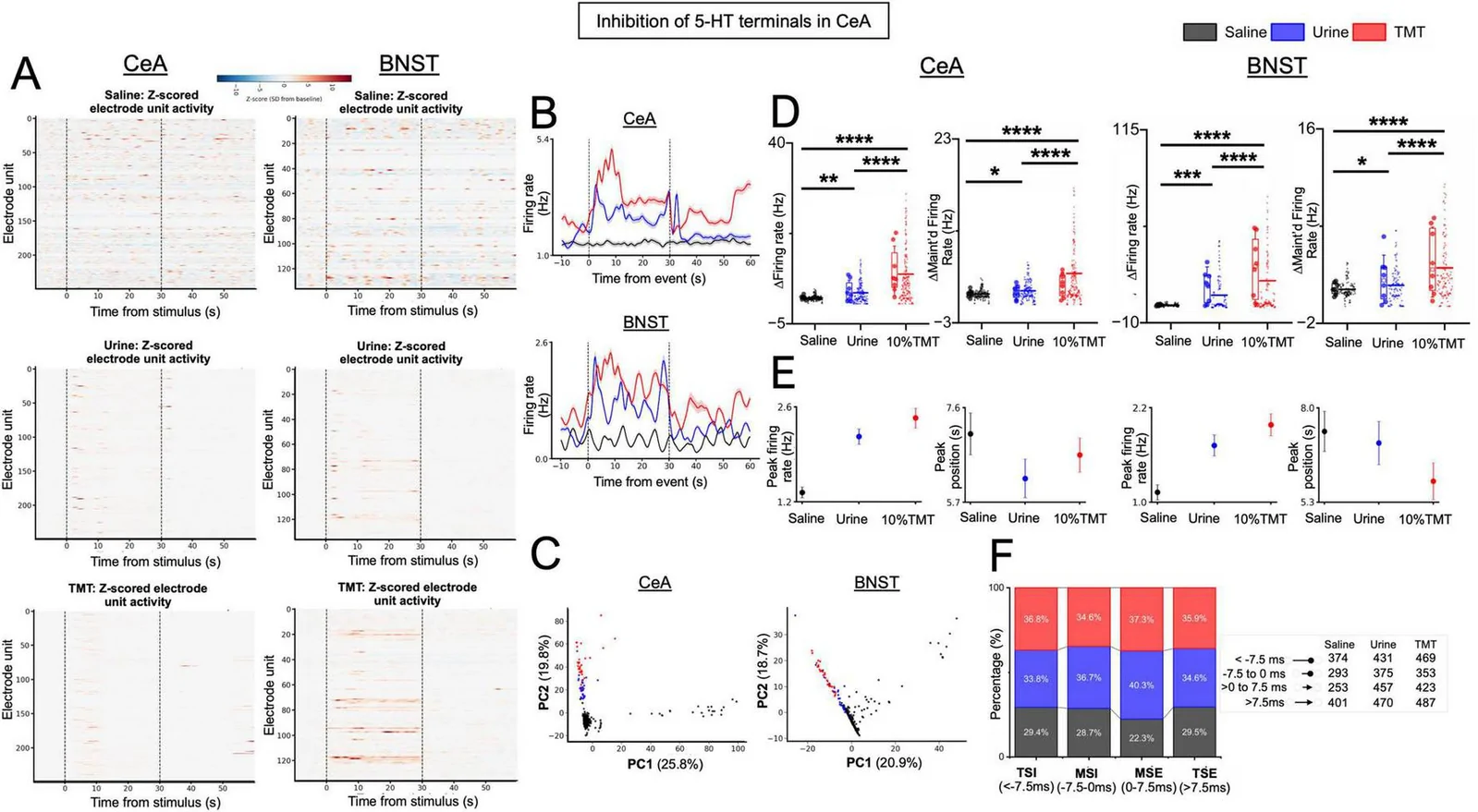
Neural coding dynamics in the BNST and CeA following inhibition of serotonergic (5-HT) terminals in the CeA. **(A)** Z-scored neural activity of all recorded units in the BNST and CeA before, during, and after odorant presentations. The scale is the same for all conditions. **(B)** Average perievent firing rate before, during (0–30 s; dashed lines indicate stimulus onset and offset), and after odorant presentation across all putative GABAergic neurons in the BNST and CeA of experimental animals. **(C)** Principal component analysis (PCA) of the firing rate of putative GABAergic units in the BNST and CeA following odorant presentations. **(D)** Firing rate and maintained firing rate of putative GABAergic units in the BNST and CeA following odorant presentations. Left: Animal-level distribution of the mean responses of putative GABAergic neurons from each animal (one point per animal). Right: Neuron-level distribution of individual GABAergic neuronal responses (one point per neuron). Animal identity was included as a random effect in the Linear mixed-effects model. {[CeA, firing rate (FR): saline vs. urine *p* = 0.0027; saline vs. 10% TMT *p* < 0.0001; urine vs. 10% TMT *p* < 0.0001; CeA, maintained FR: saline vs. urine *p* = 0.020; saline vs. 10% TMT *p* < 0.0001; urine vs. 10% TMT *p* < 0.0001] and [BNST, FR: saline vs. urine *p* = 0.00015; saline vs. 10% TMT *p* < 0.0001; urine vs. 10% TMT *p* < 0.0001; BNST, maintained FR: saline vs. urine *p* = 0.013; saline vs. 10% TMT *p* < 0.0001; urine vs. 10% TMT *p* < 0.0001]}. **(E)** Normalized peak firing rate and peak position in the BNST and CeA during odorant presentations (Peak FR, BNST: saline vs. urine *p* = 0.48; saline vs. 10% TMT *p* = 0.90; urine vs. 10% TMT *p* = 0.76; Peak FR, CeA: saline vs. urine *p* = 0.96; saline vs. 10% TMT *p* = 0.092; urine vs. 10% TMT *p* = 0.16, Peak position, BNST: saline vs. urine *p* = 0.86; saline vs. 10% TMT *p* = 0.96; urine vs. 10% TMT *p* = 0.71; Peak position, CeA: saline vs. urine *p* = 0.95; saline vs. 10% TMT *p* = 0.84; urine vs. 10% TMT *p* = 0.66). **(F)** Cross-correlogram analysis (CCG) based on the firing rate patterns between the BNST and CeA during odorant presentations. (Linear mixed model with *post hoc* model-based contrasts and correction for multiple comparisons using the Holm-Bonferroni method; *P < 0.05, **P < 0.01, ***P < 0.001, ****P < 0.0001).

## 4 Discussion

Our results suggest that activation of DRN 5-HT terminals in the CeA disrupts valence coding during stimulus presentation in both the BNST and CeA. However, after stimulus offset, valence coding is enhanced in both regions. Moreover, inhibition of DRN 5-HT terminals in the CeA facilitated valence coding in both the BNST and CeA during and after stimulus onset. The CeA has previously been shown to be a convergent site for encoding stimuli of opposite valence (Kong et al., 2025; Pignatelli and Beyeler, 2019; Kong and Zweifel, 2021). Dorsal raphe 5-HT inputs to the central amygdala reduce social rank in dominant mice and increase anxiety (Liu et al., 2026). Furthermore, injection of 5-HT into the amygdala evokes anxiogenic effects (Sengupta and Holmes, 2019). Gain- and loss-of-function experiments by Ren and his group in 2018 showed that DRN 5-HT release in the CeA promotes anxiogenic behaviors, whilst the deletion of its precursor, *TPH2* (Tryptophan hydroxylase 2) in the CeA, generates anxiolytic behaviors (Ren et al., 2018). Collateralization of DRN 5-HT inputs to the CeA has been observed in the BNST (Ren et al., 2018), demonstrating reciprocal connections between CeA/BNST and DRN. The contribution of these collaterals should be explored in future studies. Our results are consistent with these prior studies, as valence coding was impaired following activation of DRN 5-HT terminals in the CeA. Such disruption may contribute to altered valence processing, potentially generating uncertainty that could promote approach–avoidance conflict and increase anxiety-like behaviors. Future studies should investigate these neural dynamics alongside behavioral assessment to comprehensively determine their functional impact. Our results further suggest that DRN serotonergic modulation of the CeA was more critical for BNST↔CeA crosstalk than BNST modulation.

Due to its nearly ubiquitous distribution in the brain (Muzerelle et al., 2016), its bimodal modulation of valence (Nagai et al., 2020), and its status as a primary target for anxiolytics, the brain serotonergic system is crucial for valence encoding and warrants investigation. Given the amygdala’s well-established role in valence coding across multiple convergent studies (Pignatelli and Beyeler, 2019; Paton et al., 2006; Beyeler et al., 2018; O’Neill et al., 2018; Kyriazi et al., 2018), we sought to examine how the serotonergic system modulates valence encoding in the central amygdala (CeA) and its anatomically and functionally extended region, the bed nucleus of the stria terminalis (BNST), which has also been linked to valence processing (Kaouane et al., 2021; Lebow and Chen, 2016; Hammack et al., 2009; Sullivan et al., 2004; Walker and Davis, 1997). Across species, both the amygdala (Jacobs et al., 1978; Steinbusch, 1981; Asan et al., 2013; Liu et al., 2026) and BNST (Aklan et al., 2023; Garcia-Garcia et al., 2018; Zheng et al., 2024) receive dense serotonergic inputs from the principal raphe nucleus, the dorsal raphe nucleus (DRN). To investigate how the serotonergic system influences valence coding in the CeA and BNST, we performed parallel acute *in vivo* electrophysiological recordings in both regions, along with region-specific serotonergic modulation. In the amygdala, the CeA was selected for recording due to its role as the main output hub (Pignatelli and Beyeler, 2019; Duvarci and Pare, 2014). Previous studies have shown that the amygdala and BNST express distinct 5-HT receptors (Douglass et al., 2017; Sengupta et al., 2017; Asan et al., 2013; Hazra et al., 2012; Levita et al., 2004; Guo et al., 2009). For instance, 5-HT2A receptors in the CeA have been found to regulate food intake and enhance positive reinforcement (Douglass et al., 2017). Furthermore, direct infusion of a 5-HT2C receptor antagonist into the BNST was reported to abolish citalopram-induced enhancement of fear memory (Pelrine et al., 2016).

Our results further suggest that the activation of 5-HT terminals in the BNST contributes to valence coding in the BNST and CeA during stimulus presentation. Interestingly, the same modulation appears to preferentially affect only CeA-dependent valence processing following stimulus termination, suggesting temporally distinct contributions within the BNST-CeA circuit under serotonergic modulation. Contrastingly, the inhibition of 5-HT terminals in the BNST supports a model in which serotonergic suppression is critical for valence coding in the BNST, whereas coding in the CeA may be impaired, whether during stimulus presentation or following stimulus termination. Several studies have used neural firing responses to negative and positive valence stimuli as a metric for defining valence coding (Beyeler et al., 2016; Paton et al., 2006; Schoenbaum et al., 1999; Shabel and Janak, 2009). A comparable response of neurons to positive and negative valence stimuli supports an arousal response (Pignatelli and Beyeler, 2019), in which these neurons may preferentially signal arousal rather than discriminate valence. However, valence can be conceptualized as differential responsiveness to positive and negative stimuli (Paton et al., 2006; Beyeler et al., 2018; O’Neill et al., 2018; Kyriazi et al., 2018). Here, neurons still encode valence as they exhibit a greater response to one valence type.

Using optogenetic manipulation, a previous study found that 5-HT release throughout the entire BNST elicits anxiogenic effects (Marcinkiewcz et al., 2016). However, another line of evidence suggests that 5-HT activation in the dorsal BNST reduces anxiety in an environment more likely to induce anxiety and vice versa (Garcia-Garcia et al., 2018). Contrastingly, Kim et al. (2013) found that direct inhibition of the dorsal BNST and its oval division reduces anxiety. The differential expression of high-affinity 5-HT1A (inhibitory) and low-affinity 5-HT2C (excitatory) receptors in the dorsal and ventral BNST, respectively (Hammack et al., 2009), could contribute to these varying outcomes. Our results suggest that activation of DRN 5-HT terminals in the lateral BNST is more likely to transiently inhibit oval BNST cells, which may reduce anxiety during the period of stimulus presentation when valence is discriminated but not following stimulus termination. The ability of BNST and amygdala circuits to accurately process and discriminate valence can limit the activation of the hypothalamic-pituitary-adrenal (HPA) axis, preventing the overgeneralization of fear and induction of chronic stress. Our results also suggest that 5-HT suppression in the lateral BNST enhances valence coding only in the BNST, but not in the CeA, probably because it reduces the BNST’s output to downstream targets without necessarily suppressing its intrinsic ability. The lateral BNST expresses both 5-HT1A (inhibitory) and 5-HT2C (excitatory) receptors (Guo et al., 2009). The activation of DRN 5-HT terminals in the BNST likely balances the output to the CeA, whereas the inactivation of 5-HT terminals likely dampens the BNST→CeA crosstalk.

Urethane has been reported to attenuate serotonergic neurotransmission and enhance the effects of antagonists relative to awake states (Dringenberg and Vanderwolf, 1995), while also increasing basal firing in 5-HT neurons (McCardle and Gartside, 2012). In addition, serotonergic neurons can modulate anesthetic depth (Li et al., 2021), creating a bidirectional confound in which anesthesia and serotonergic activity may influence one another. As such, future studies in freely behaving awake rodents are warranted to further investigate these dynamics and assess their functional impact. Although VGLUT-3, the predominant vesicular glutamate transporter expressed in the DRN, colocalizes with only a small subset of serotonergic neurons in the lateral wings (Hioki et al., 2010), future studies should investigate how interactions with other neurotransmitter systems, including glutamatergic and GABAergic signaling, influence valence-related neuronal dynamics within these circuits. Although local intracranial infusion of the DREADD agonist 21 was used to selectively modulate DREADD signaling at DRN 5-HT terminals, the present study did not directly quantify changes in local serotonin. Future studies combining this approach with direct measurements of serotonin dynamics or additional ligand-control experiments would further strengthen these findings. Although female urine and TMT are well-established ethologically relevant appetitive and aversive stimuli, respectively, the present design cannot completely disentangle neural responses to valence from responses related to odor identity or stimulus salience. Future studies incorporating multiple stimuli within each valence category, behavioral validation under the recording conditions, and intensity-matched odors will be important for determining the extent to which serotonergic modulation generalizes across valence categories.

## 5 Conclusion

Our results suggest that the dorsal raphe serotonergic system dynamically processes valence within the BNST-CeA circuit in a temporally and regionally distinct manner. During stimulus presentation, activation of 5-HT terminals in the BNST facilitated valence coding across both regions. Following stimulus offset, this modulation preferentially engaged only CeA-dependent valence coding. In contrast, inhibition of DRN 5-HT terminals in the BNST supported local valence coding, with limited effects on CeA-dependent processing. Within the CeA, activation of DRN 5-HT terminals disrupts valence coding in both CeA and BNST. However, following stimulus offset, valence coding was facilitated in both brain regions. In contrast, 5-HT inhibition in the CeA enhanced valence coding in both brain regions during and after stimulus presentation.

These findings may reflect serotonin’s role in modulating the temporal dynamics and fidelity of valence encoding within the BNST-CeA circuit. Such modulation may contribute to stimulus evaluation and be involved in neural processes underlying the resolution of approach-avoidance conflicts and the regulation of adaptive behaviors. As such, its dysregulation could contribute as a circuit substrate for altered emotional processing associated with affective disorders.

## Data Availability

The raw data supporting the conclusions of this article will be made available by the authors, without undue reservation.

## Supplementary material

Supplementary Figure 1 Viral vector expression and *post hoc* verification of recording sites. (**A**) Bilateral injection (1 μL on each side) of anterograde AAV-hSyn-DIO-hM3D(Gq)-mcherry or anterograde AAV-hSyn-DIO-hM4D(Gi)-mcherry in the dorsal raphe nucleus (DRN). (**B**) Anatomical verification of central amygdala (CeA) recording electrode placement via rhodamine track. (**C**) Anatomical verification of bed nucleus of stria terminalis (BNST) recording electrode placement via fluorogold track. (**D**) Cell body innervation/terminals of labeled DRN serotonergic neurons expressing either hM3D(Gq) or hM4D(Gi) in the CeA. (**E**) Cell body innervation/terminals of labeled DRN serotonergic neurons expressing either hM3D(Gq) or hM4D(Gi) in the BNST.

Supplementary Figure 2 *Post hoc* characterization of putative glutamatergic and GABAergic neurons. Marginal histogram showing the clustering of glutamatergic (blue circles) and GABAergic units (purple triangles) sampled in the BNST and CeA by trough-to-peak slope and peak width at half height for experimental animals with (**A**) activation of serotonergic (5-HT) terminals in the BNST, (**B**) inhibition of 5-HT terminals in the BNST, (**C**) activation of 5-HT terminals in the CeA, and (**D**) inhibition of 5-HT terminals in the CeA. (**E**) Sample waveform and autocorrelogram of putative glutamatergic units shown in a 1 ms window and 50 ms bins, respectively. (**F**) Sample waveform and autocorrelogram of putative GABAergic units shown in a 1 ms window and 50 ms bins, respectively.

Supplementary Figure 3 Interspike interval and firing regularity of putative GABAergic units in the BNST and CeA during odorant presentations. Interspike interval (ISI) and firing regularity (CV) of putative GABAergic units in the BNST and CeA following odorant presentations for experimental animals with panel (**A**) activation of serotonergic (5-HT) terminals in the BNST [(BNST, interspike interval (ISI): saline vs. urine *p* < 0.0001; saline vs. 10% TMT *p* < 0.0001; urine vs. 10% TMT *p* = 0.031; BNST, firing regularity (CV): saline vs. urine *p* < 0.0001; saline vs. 10% TMT *p* < 0.0001; urine vs. 10% TMT *p* = 0.51] and [(CeA, ISI: saline vs. urine *p* = 0.17; saline vs. 10% TMT *p* < 0.0001; urine vs. 10% TMT *p* < 0.0001; CeA, CV: saline vs. urine *p* = 0.0011; saline vs. 10% TMT *p* = 0.018; urine vs. 10% TMT: *p* = 0.34)]. (**B**) inhibition of 5-HT terminals in the BNST [(BNST, ISI: saline vs. urine *p* < 0.0001; saline vs. 10% TMT *p* < 0.0001; urine vs. 10% TMT *p* = 0.0048; BNST, CV: saline vs. urine *p* < 0.0001; saline vs. 10% TMT *p* = 0.00029; urine vs. 10% TMT *p* = 0.61) and (CeA, ISI: saline vs. urine *p* < 0.0001; saline vs. 10% TMT *p* < 0.0001; urine vs. 10% TMT *p* = 0.66; CeA, CV: saline vs. urine *p* = 0.0038; saline vs. 10% TMT *p* = 0.018; urine vs. 10% TMT *p* = 0.58)]. (**C**) activation of 5-HT terminals in the CeA [(CeA, ISI: saline vs. urine *p* < 0.0001; saline vs. 10% TMT *p* < 0.0001; urine vs. 10% TMT *p* = 0.14; CeA, CV: saline vs. urine *p* = 0.0044; saline vs. 10% TMT: *p* = 0.27; urine vs. 10% TMT: *p* = 0.00011) and (BNST, ISI: saline vs. urine *p* < 0.0001; saline vs. 10% TMT *p* < 0.0001; urine vs. 10% TMT *p* = 0.19; BNST, CV: saline vs. urine *p* = 0.40; saline vs. 10% TMT *p* = 0.32; urine vs. 10% TMT: *p* = 0.88)]. (**D**) inhibition of 5-HT terminals in the CeA [(CeA, ISI: saline vs. urine *p* < 0.0001; saline vs. 10% TMT *p* < 0.0001; urine vs. 10% TMT *p* = 0.00058; CeA, CV: saline vs. urine *p* = 0.013; saline vs. 10% TMT *p* = 0.65; urine vs. 10% TMT *p* = 0.0034) and (BNST, ISI: saline vs. urine *p* < 0.0001; saline vs. 10% TMT *p* < 0.0001; urine vs. 10% TMT *p* = 0.0038; BNST, CV: saline vs. urine *p* = 0.11; saline vs. 10% TMT *p* = 0.0026; urine vs. 10% TMT *p* < 0.0001)] (Linear mixed model with *post hoc* model-based contrasts and correction for multiple comparisons using the Holm-Bonferroni method; **P* < 0.05, ***P* < 0.01, ****P* < 0.001, *****P* < 0.0001).

Supplementary Figure 4 Burst activity of putative GABAergic units in the BNST and CeA during odorant presentations. Burst per minute of putative GABAergic units in the BNST and CeA following odorant presentations for experimental animals with panel (**A**) activation of serotonergic (5-HT) terminals in the BNST (BNST: saline vs. urine *p* = 0.98; saline vs. 10% TMT *p* = 0.090; urine vs. 10% TMT *p* = 0.084 and CeA: saline vs. urine *p* = 0.072; saline vs. 10% TMT *p* = 0.48; urine vs. 10% TMT *p* = 0.013). (**B**) Inhibition of 5-HT terminals in the BNST (BNST: saline vs. urine *p* = 0.011; saline vs. 10% TMT *p* = 0.0044; urine vs. 10% TMT *p* = 0.76 and CeA: saline vs. urine *p* = 0.061; saline vs. 10% TMT *p* = 0.079; urine vs. 10% TMT *p* = 0.00061). (**C**) activation of 5-HT terminals in the CeA (CeA: saline vs. urine *p* = 0.020; saline vs. 10% TMT *p* = 0.017; urine vs. 10% TMT *p* < 0.0001 and BNST: saline vs. urine *p* = 0.063; saline vs. 10% TMT *p* = 0.22; urine vs. 10% TMT *p* = 0.53). (**D**) inhibition of 5-HT terminals in the CeA (CeA: saline vs. urine *p* = 0.27; saline vs. 10% TMT *p* < 0.0001; urine vs. 10% TMT *p* = 0.00091 and BNST: saline vs. urine *p* = 0.28; saline vs. 10% TMT *p* = 0.16; urine vs. 10% TMT *p* = 0.014) (Linear mixed model with *post hoc* model-based contrasts and correction for multiple comparisons using the Holm-Bonferroni method; **P* < 0.05, ***P* < 0.01, ****P* < 0.001, *****P* < 0.0001).

Supplementary Figure 5 Percentage of spikes in burst activity of putative GABAergic units in the BNST and CeA during odorant presentations. Percentage of spikes in burst activity of putative GABAergic units in the BNST and CeA following odorant presentations for experimental animals with (**A**) activation of serotonergic (5-HT) terminals in the BNST (BNST: saline vs. urine *p* < 0.0001; saline vs. 10% TMT *p* < 0.0001; urine vs. 10% TMT *p* = 0.89 and CeA: saline vs. urine *p* = 0.0027; saline vs. 10% TMT *p* = 0.11; urine vs. 10% TMT *p* = 0.16). (**B**) Inhibition of 5-HT terminals in the BNST (BNST: saline vs. urine *p* = 0.30; saline vs. 10% TMT *p* = 0.33; urine vs. 10% TMT *p* = 0.048 and CeA: saline vs. urine *p* < 0.0001; saline vs. 10% TMT *p* = 0.00030; urine vs. 10% TMT *p* = 0.38). (**C**) Activation of 5-HT terminals in the CeA (CeA: saline vs. urine *p* = 0.027; saline vs. 10% TMT *p* < 0.0001; urine vs. 10% TMT *p* = 0.0073 and BNST: saline vs. urine *p* = 0.00049; saline vs. 10% TMT *p* = 0.67; urine vs. 10% TMT *p* = 0.0021). (**D**) Inhibition of 5-HT terminals in the CeA (CeA: saline vs. urine *p* = 0.73; saline vs. 10% TMT *p* = 0.070; urine vs. 10% TMT *p* = 0.15 and BNST: saline vs. urine *p* = 0.00065; saline vs. 10% TMT *p* = 0.029; urine vs. 10% TMT *p* = 0.19) (Linear mixed model with *post hoc* model-based contrasts and correction for multiple comparisons using the Holm-Bonferroni method; **P* < 0.05, ***P* < 0.01, ****P* < 0.001, *****P* < 0.0001).

Supplementary Figure 6 Burst duration of putative GABAergic units in the BNST and CeA during odorant presentations. Burst duration of putative GABAergic units in the BNST and CeA following odorant presentations for experimental animals with panel **(A)** activation of serotonergic (5-HT) terminals in the BNST (BNST: saline vs. urine *p* < 0.0001; saline vs. 10% TMT *p* < 0.0001; urine vs. 10% TMT *p* = 0.37 and CeA: saline vs. urine *p* < 0.0001; saline vs. 10% TMT *p* < 0.0001; urine vs. 10% TMT *p* = 0.20). **(B)** Inhibition of 5-HT terminals in the BNST (BNST: saline vs. urine *p* < 0.0001; saline vs. 10% TMT *p* < 0.0001; urine vs. 10% TMT *p* = 0.61 and CeA: saline vs. urine *p* < 0.0001; saline vs. 10% TMT *p* < 0.0001; urine vs. 10% TMT *p* = 0.67). **(C)** Activation of 5-HT terminals in the CeA (CeA: saline vs. urine *p* < 0.0001; saline vs. 10% TMT *p* < 0.0001; urine vs. 10% TMT *p* = 0.18 and BNST: saline vs. urine *p* < 0.0001; saline vs. 10% TMT *p* < 0.0001; urine vs. 10% TMT *p* = 0.18). **(D)** Inhibition of 5-HT terminals in the CeA (CeA: saline vs. urine *p* < 0.0001; saline vs. 10% TMT *p* < 0.0001; urine vs. 10% TMT *p* = 0.23 and BNST: saline vs. urine *p* < 0.0001; saline vs. 10% TMT *p* < 0.0001; urine vs. 10% TMT *p* = 0.76) (Linear mixed model with post-hoc model-based contrasts and correction for multiple comparisons using the Holm-Bonferroni method; *****P* < 0.0001).

Supplementary Figure 7 Principal component analysis (PCA) of the electrophysiological endpoints in the BNST and CeA following activation of 5-HT terminals in the BNST. PCA of putative GABAergic units in the BNST and CeA following activation of 5-HT terminals in the BNST during odorant presentations for the (**A**) firing rate, (**B**) mean ISI, (**C**) coefficient of variation (CV) of the interspike interval (ISI), and (D) burst rate/burstiness.

Supplementary Figure 8 Principal component analysis (PCA) of the electrophysiological endpoints in the BNST and CeA following inhibition of 5-HT terminals in the BNST. PCA of putative GABAergic units in the BNST and CeA following inhibition of 5-HT terminals in the BNST during odorant presentations for the (**A**) firing rate, (**B**) mean ISI, (**C**) coefficient of variation (CV) of the interspike interval (ISI), and (**D**) burst rate/burstiness.

Supplementary Figure 9 Principal component analysis (PCA) of the electrophysiological endpoints in the BNST and CeA following activation of 5-HT terminals in the CeA. PCA of putative GABAergic units in the BNST and CeA following activation of 5-HT terminals in the CeA during odorant presentations for the (**A**) firing rate, (**B**) mean ISI, (**C**) coefficient of variation (CV) of the interspike interval (ISI), and (**D**) burst rate/burstiness.

Supplementary Figure 10 Principal component analysis (PCA) of the electrophysiological endpoints in the BNST and CeA following inhibition of 5-HT terminals in the CeA. PCA of putative GABAergic units in the BNST and CeA following inhibition of 5-HT terminals in the CeA during odorant presentations for the (**A**) firing rate, (**B**) mean ISI, (**C**) coefficient of variation (CV) of the interspike interval (ISI), and (**D**) burst rate/burstiness.

Supplementary Table 1 Summary of fixed and random effects from the linear mixed model (LMM).
