# Supplementary material for: Serotonergic control of valence processing in amygdalo- striaterminalis circuits
Source: Front Neural Circuits. 2026 Oct 26;20:1906095. doi: 10.3389/fncir.2026.1906095 (PMC13745565; doi:10.3389/fncir.2026.1906095)
Supplement: Supplementary file 1 [file Table_1.pdf]

Parameter	Group	Fixed & random effects	Estimate (β)	SE	z	p-value	95% CI (2.5%, 97.5%)
Firing rate (FR, Hz)	Activation of 5-HT terminals in BNST (*record in BNST*)	Saline (ref. mean)	1.315	0.082	15.963	0.000	1.154, 1.477
		Urine vs Saline	0.478	0.089	5.342	0.000	0.302, 0.653
		TMT vs Saline	0.761	0.089	8.515	0.000	0.586, 0.936
		Animal ID (random intercept)	0.497	0.119			
	Activation of 5-HT terminals in BNST (*record in CeA*)	Saline (ref. mean)	0.912	0.024	38.497	0.000	0.865, 0.958
		Urine vs Saline	0.070	0.020	3.555	0.000	0.031, 0.108
		TMT vs Saline	0.154	0.020	7.777	0.000	0.115, 0.193
		Animal ID (random intercept)	0.070	0.056			
	Inhibition of 5-HT terminals in BNST (*record in BNST*)	Saline (ref. mean)	1.573	0.267	5.900	0.000	1.050, 2.095
		Urine vs Saline	1.336	0.310	4.305	0.000	0.728, 1.944
		TMT vs Saline	2.626	0.292	8.986	0.000	2.053, 3.199
		Animal ID (random intercept)	2.862	0.449			
	Inhibition of 5-HT terminals in BNST (*record in CeA*)	Saline (ref. mean)	1.444	0.451	3.202	0.001	0.560, 2.328
		Urine vs Saline	3.436	0.463	7.417	0.000	2.528, 4.344
		TMT vs Saline	2.820	0.460	6.132	0.000	1.918, 3.721
		Animal ID (random intercept)	10.903	0.752			
	Activation of 5-HT terminals in CeA (*record in BNST*)	Saline (ref. mean)	1.056	0.045	23.341	0.000	0.967, 1.144
		Urine vs Saline	0.479	0.045	10.625	0.000	0.391, 0.567
		TMT vs Saline	0.460	0.045	10.165	0.000	0.371, 0.548
		Animal ID (random intercept)	0.103	0.077			
	Activation of 5-HT terminals in CeA (*record in CeA*)	Saline (ref. mean)	1.296	0.094	13.844	0.000	1.113, 1.480
		Urine vs Saline	0.652	0.117	5.563	0.000	0.422, 0.881
		TMT vs Saline	0.483	0.112	4.312	0.000	0.263, 0.702
		Animal ID (random intercept)	0.257	0.117			

	Inhibition of 5-HT terminals in CeA (*record in BNST*)	Saline (ref. mean)	1.679	1.429	1.175	0.240	-1.122, 4.479
		Urine vs Saline	6.867	1.795	3.825	0.000	3.349, 10.386
		TMT vs Saline	15.789	1.794	8.800	0.000	12.273, 19.306
		Animal ID (random intercept)	62.464	1.619			
	Inhibition of 5-HT terminals in CeA (*record in CeA*)	Saline (ref. mean)	1.517	0.338	4.484	0.000	0.854, 2.179
		Urine vs Saline	1.412	0.468	3.017	0.003	0.495, 2.329
		TMT vs Saline	5.840	0.444	13.154	0.000	4.970, 6.710
		Animal ID (random intercept)	3.096	0.403			
Maintained firing rate (Maint'd FR, Hz)	Activation of 5-HT terminals in BNST (*record in BNST*)	Saline (ref. mean)	1.289	0.040	31.891	0.000	1.210, 1.368
		Urine vs Saline	0.055	0.033	1.693	0.090	-0.009, 0.120
		TMT vs Saline	0.089	0.033	2.734	0.006	0.025, 0.153
		Animal ID (random intercept)	0.204	0.099			
	Activation of 5-HT terminals in BNST (*record in CeA*)	Saline (ref. mean)	0.929	0.022	41.385	0.000	0.885, 0.973
		Urine vs Saline	-0.095	0.014	-6.607	0.000	-0.123, -0.067
		TMT vs Saline	-0.008	0.014	-0.571	0.568	-0.037, 0.020
		Animal ID (random intercept)	0.076				
	Inhibition of 5-HT terminals in BNST (*record in BNST*)	Saline (ref. mean)	2.094	1.462	1.432	0.152	-0.772, 4.959
		Urine vs Saline	10.386	1.783	5.825	0.000	6.891, 13.880
		TMT vs Saline	4.342	1.790	2.425	0.015	0.833, 7.852
		Animal ID (random intercept)	54.236	1.740			
	Inhibition of 5-HT terminals in BNST (*record in CeA*)	Saline (ref. mean)	1.539	0.403	3.816	0.000	0.748, 2.329
		Urine vs Saline	2.317	0.387	5.993	0.000	1.559, 3.075
		TMT vs Saline	2.019	0.384	5.262	0.000	1.267, 2.771
		Animal ID (random intercept)	10.505	0.793			
	Activation of 5-HT terminals in CeA	Saline (ref. mean)	1.066	0.041	26.165	0.000	0.986, 1.146
		Urine vs Saline	0.098	0.036	2.724	0.006	0.027, 0.168

	(*record in BNST*)	TMT vs Saline	0.352	0.036	9.859	0.000	0.282, 0.422
		Animal ID (random intercept)	0.101	0.086			
	Activation of 5-HT terminals in CeA (*record in CeA*)	Saline (ref. mean)	1.249	0.051	24.552	0.000	1.149, 1.349
		Urine vs Saline	0.001	0.057	0.022	0.983	-0.110, 0.113
		TMT vs Saline	0.157	0.057	2.754	0.006	0.045, 0.269
		Animal ID (random intercept)	0.096				
	Inhibition of 5-HT terminals in CeA (*record in BNST*)	Saline (ref. mean)	1.477	0.181	8.176	0.000	1.123, 1.831
		Urine vs Saline	0.522	0.210	2.485	0.013	0.110, 0.934
		TMT vs Saline	1.884	0.203	9.280	0.000	1.486, 2.282
		Animal ID (random intercept)	1.638	0.261			
	Inhibition of 5-HT terminals in CeA (*record in CeA*)	Saline (ref. mean)	1.433	0.195	7.349	0.000	1.051, 1.815
		Urine vs Saline	0.602	0.258	2.336	0.019	0.097, 1.107
		TMT vs Saline	2.801	0.248	11.286	0.000	2.315, 3.287
		Animal ID (random intercept)	1.589	0.222			
Interspike interval (ISI, ms)	Activation of 5-HT terminals in BNST (*record in BNST*)	Saline (ref. mean)	0.749	0.023	31.971	0.000	0.703, 0.794
		Urine vs Saline	-0.159	0.024	-6.505	0.000	-0.207, -0.111
		TMT vs Saline	-0.212	0.025	-8.622	0.000	-0.260, -0.164
		Animal ID (random intercept)	0.047	0.037			
	Activation of 5-HT terminals in BNST (*record in CeA*)	Saline (ref. mean)	1.118	0.032	34.573	0.000	1.055, 1.181
		Urine vs Saline	-0.038	0.027	-1.387	0.165	-0.091, 0.016
		TMT vs Saline	-0.162	0.027	-5.956	0.000	-0.216, -0.109
		Animal ID (random intercept)	0.126	0.073			
	Inhibition of 5-HT terminals in BNST (*record in BNST*)	Saline (ref. mean)	0.788	0.027	29.018	0.000	0.735, 0.841
		Urine vs Saline	-0.434	0.025	-17.32	0.000	-0.483, -0.385
		TMT vs Saline	-0.506	0.026	-19.82	0.000	-0.556, -0.456
		Animal ID	0.042	0.052			

		(random intercept)					
	Inhibition of 5-HT terminals in BNST (*record in CeA*)	Saline (ref. mean)	0.887	0.044	20.350	0.000	0.802, 0.973
		Urine vs Saline	-0.339	0.042	-8.134	0.000	-0.421, -0.258
		TMT vs Saline	-0.321	0.042	-7.659	0.000	-0.403, -0.239
		Animal ID (random intercept)	0.123	0.079			
	Activation of 5-HT terminals in CeA (*record in BNST*)	Saline (ref. mean)	0.974	0.027	36.362	0.000	0.921, 1.026
		Urine vs Saline	-0.331	0.021	-15.90	0.000	-0.372, -0.290
		TMT vs Saline	-0.304	0.020	-14.99	0.000	-0.344, -0.264
		Animal ID (random intercept)	0.049				
	Activation of 5-HT terminals in CeA (*record in CeA*)	Saline (ref. mean)	0.843	0.033	25.480	0.000	0.778, 0.908
		Urine vs Saline	-0.268	0.030	-8.930	0.000	-0.327, -0.209
		TMT vs Saline	-0.224	0.030	-7.518	0.000	-0.282, -0.166
		Animal ID (random intercept)	0.066	0.066			
	Inhibition of 5-HT terminals in CeA (*record in BNST*)	Saline (ref. mean)	0.716	0.030	24.123	0.000	0.658, 0.774
		Urine vs Saline	-0.354	0.023	-15.25	0.000	-0.400, -0.309
		TMT vs Saline	-0.421	0.023	-18.03	0.000	-0.467, -0.376
		Animal ID (random intercept)	0.077	0.076			
	Inhibition of 5-HT terminals in CeA (*record in CeA*)	Saline (ref. mean)	0.753	0.023	33.443	0.000	0.709, 0.797
		Urine vs Saline	-0.398	0.023	-16.95	0.000	-0.444, -0.352
		TMT vs Saline	-0.479	0.023	-20.45	0.000	-0.524, -0.433
		Animal ID (random intercept)	0.048	0.035			
Coefficient of variation (CV)	Activation of 5-HT terminals in BNST (*record in BNST*)	Saline (ref. mean)	1.010	0.021	48.052	0.000	0.969, 1.051
		Urine vs Saline	-0.135	0.024	-5.608	0.000	-0.182, -0.088
		TMT vs Saline	-0.120	0.024	-4.963	0.000	-0.167, -0.072
		Animal ID (random intercept)	0.028	0.027			
		Saline (ref. mean)	0.995	0.009	109.031	0.000	0.977, 1.013

	Activation of 5-HT terminals in BNST (*record in CeA*)	Urine vs Saline	-0.039	0.012	-3.282	0.001	-0.062, -0.016
		TMT vs Saline	-0.028	0.012	-2.376	0.017	-0.050, -0.005
		Animal ID (random intercept)	0.003	0.010			
	Inhibition of 5-HT terminals in BNST (*record in BNST*)	Saline (ref. mean)	1.050	0.035	30.309	0.000	0.982, 1.118
		Urine vs Saline	-0.141	0.034	-4.162	0.000	-0.208, -0.075
		TMT vs Saline	-0.124	0.034	-3.666	0.000	-0.191, -0.058
		Animal ID (random intercept)	0.058	0.063			
	Inhibition of 5-HT terminals in BNST (*record in CeA*)	Saline (ref. mean)	1.072	0.043	24.851	0.000	0.987, 1.157
		Urine vs Saline	-0.133	0.046	-2.915	0.004	-0.223, -0.044
		TMT vs Saline	-0.109	0.046	-2.377	0.017	-0.199, -0.019
		Animal ID (random intercept)	0.091	0.063			
	Activation of 5-HT terminals in CeA (*record in BNST*)	Saline (ref. mean)	0.969	0.016	62.393	0.000	0.939, 1.000
		Urine vs Saline	-0.015	0.018	-0.847	0.397	-0.050, 0.020
		TMT vs Saline	-0.018	0.018	-0.989	0.323	-0.052, 0.017
		Animal ID (random intercept)	0.008	0.019			
	Activation of 5-HT terminals in CeA (*record in CeA*)	Saline (ref. mean)	1.018	0.017	61.213	0.000	0.985, 1.050
		Urine vs Saline	0.057	0.020	2.871	0.004	0.018, 0.096
		TMT vs Saline	-0.022	0.020	-1.096	0.273	-0.062, 0.017
		Animal ID (random intercept)	0.008	0.021			
	Inhibition of 5-HT terminals in CeA (*record in BNST*)	Saline (ref. mean)	1.058	0.047	22.522	0.000	0.966, 1.150
		Urine vs Saline	-0.085	0.053	-1.587	0.112	-0.189, 0.020
		TMT vs Saline	0.163	0.054	3.035	0.002	0.058, 0.269
		Animal ID (random intercept)	0.090	0.061			
	Inhibition of 5-HT terminals in CeA (*record in CeA*)	Saline (ref. mean)	1.075	0.034	31.905	0.000	1.009, 1.141
		Urine vs Saline	-0.106	0.043	-2.481	0.013	-0.190, -0.022
		TMT vs Saline	0.020	0.043	0.458	0.647	-0.065, 0.104

		Animal ID (random intercept)	0.044	0.032			
Burst per minute	Activation of 5-HT terminals in BNST (*record in BNST*)	Saline (ref. mean)	1.364	0.125	10.916	0.000	1.119, 1.608
		Urine vs Saline	0.004	0.158	0.023	0.982	-0.307, 0.314
		TMT vs Saline	-0.271	0.159	-1.700	0.089	-0.583, 0.041
		Animal ID (random intercept)	0.387	0.117			
	Activation of 5-HT terminals in BNST (*record in CeA*)	Saline (ref. mean)	0.908	0.073	12.465	0.000	0.765, 1.051
		Urine vs Saline	-0.165	0.092	-1.803	0.071	-0.345, 0.014
		TMT vs Saline	0.065	0.092	0.710	0.478	-0.115, 0.244
		Animal ID (random intercept)	0.196	0.074			
	Inhibition of 5-HT terminals in BNST (*record in BNST*)	Saline (ref. mean)	1.017	0.436	2.329	0.020	0.161, 1.872
		Urine vs Saline	1.511	0.585	2.585	0.010	0.366, 2.657
		TMT vs Saline	1.687	0.580	2.909	0.004	0.550, 2.823
		Animal ID (random intercept)	0.800	0.340			
	Inhibition of 5-HT terminals in BNST (*record in CeA*)	Saline (ref. mean)	0.744	0.278	2.676	0.007	0.199, 1.289
		Urine vs Saline	0.730	0.386	1.892	0.058	-0.026, 1.486
		TMT vs Saline	-0.750	0.424	-1.770	0.077	-1.581, 0.080
		Animal ID (random intercept)	0.096	0.194			
	Activation of 5-HT terminals in CeA (*record in BNST*)	Saline (ref. mean)	1.118	0.222	5.028	0.000	0.682, 1.554
		Urine vs Saline	0.539	0.288	1.869	0.062	-0.026, 1.104
		TMT vs Saline	0.358	0.291	1.228	0.219	-0.213, 0.929
		Animal ID (random intercept)	0.538	0.203			
	Activation of 5-HT terminals in CeA (*record in CeA*)	Saline (ref. mean)	1.283	0.212	6.063	0.000	0.869, 1.698
		Urine vs Saline	0.690	0.294	2.343	0.019	0.113, 1.267
		TMT vs Saline	-0.719	0.299	-2.402	0.016	-1.306, -0.132
		Animal ID (random intercept)	0.015	0.204			

	Inhibition of 5-HT terminals in CeA (*record in BNST*)	Saline (ref. mean)	0.857	0.192	4.454	0.000	0.480, 1.235
		Urine vs Saline	-0.250	0.229	-1.091	0.275	-0.699, 0.199
		TMT vs Saline	0.328	0.230	1.427	0.154	-0.123, 0.779
		Animal ID (random intercept)	0.536	0.228			
	Inhibition of 5-HT terminals in CeA (*record in CeA*)	Saline (ref. mean)	1.229	0.440	2.791	0.005	0.366, 2.092
		Urine vs Saline	0.656	0.589	1.113	0.266	-0.498, 1.810
		TMT vs Saline	2.641	0.576	4.582	0.000	1.511, 3.770
		Animal ID (random intercept)	2.243	0.457			
% spikes in burst	Activation of 5-HT terminals in BNST (*record in BNST*)	Saline (ref. mean)	1.203	0.076	15.789	0.000	1.054, 1.353
		Urine vs Saline	-0.514	0.098	-5.262	0.000	-0.705, -0.322
		TMT vs Saline	-0.527	0.097	-5.465	0.000	-0.717, -0.338
		Animal ID (random intercept)	0.165	0.080			
	Activation of 5-HT terminals in BNST (*record in CeA*)	Saline (ref. mean)	1.027	0.071	14.473	0.000	0.888, 1.166
		Urine vs Saline	-0.281	0.093	-3.012	0.003	-0.464, -0.098
		TMT vs Saline	-0.149	0.094	-1.591	0.112	-0.333, 0.035
		Animal ID (random intercept)	0.127	0.064			
	Inhibition of 5-HT terminals in BNST (*record in BNST*)	Saline (ref. mean)	0.961	0.176	5.445	0.000	0.615, 1.307
		Urine vs Saline	0.244	0.237	1.031	0.303	-0.220, 0.708
		TMT vs Saline	-0.231	0.238	-0.971	0.331	-0.698, 0.236
		Animal ID (random intercept)	0.126	0.136			
	Inhibition of 5-HT terminals in BNST (*record in CeA*)	Saline (ref. mean)	0.830	0.103	8.073	0.000	0.628, 1.031
		Urine vs Saline	-0.676	0.151	-4.470	0.000	-0.972, -0.380
		TMT vs Saline	-0.540	0.145	-3.721	0.000	-0.824, -0.255
		Animal ID (random intercept)	0.020	0.071			
	Activation of 5-HT terminals in CeA	Saline (ref. mean)	1.136	0.104	10.906	0.000	0.932, 1.340
		Urine vs Saline	-0.467	0.132	-3.540	0.000	-0.725, -0.208

	(*record in BNST*)	TMT vs Saline	-0.056	0.129	-0.429	0.668	-0.309, 0.198
		Animal ID (random intercept)	0.186	0.110			
	Activation of 5-HT terminals in CeA (*record in CeA*)	Saline (ref. mean)	1.164	0.094	12.370	0.000	0.980, 1.348
		Urine vs Saline	-0.290	0.130	-2.232	0.026	-0.544, -0.035
		TMT vs Saline	-0.640	0.130	-4.933	0.000	-0.894, -0.386
		Animal ID (random intercept)	0.028	0.060			
	Inhibition of 5-HT terminals in CeA (*record in BNST*)	Saline (ref. mean)	0.781	0.103	7.597	0.000	0.580, 0.983
		Urine vs Saline	-0.436	0.125	-3.492	0.000	-0.681, -0.191
		TMT vs Saline	-0.273	0.124	-2.209	0.027	-0.516, -0.031
		Animal ID (random intercept)	0.138	0.125			
	Inhibition of 5-HT terminals in CeA (*record in CeA*)	Saline (ref. mean)	0.927	0.156	5.947	0.000	0.621, 1.232
		Urine vs Saline	0.073	0.211	0.347	0.728	-0.340, 0.487
		TMT vs Saline	0.382	0.209	1.823	0.068	-0.029, 0.792
		Animal ID (random intercept)	0.201	0.128			
Burst duration (s)	Activation of 5-HT terminals in BNST (*record in BNST*)	Saline (ref. mean)	0.903	0.048	18.815	0.000	0.809, 0.997
		Urine vs Saline	-0.341	0.053	-6.460	0.000	-0.445, -0.238
		TMT vs Saline	-0.389	0.053	-7.374	0.000	-0.492, -0.285
		Animal ID (random intercept)	0.121	0.064			
	Activation of 5-HT terminals in BNST (*record in CeA*)	Saline (ref. mean)	1.050	0.041	25.871	0.000	0.971, 1.130
		Urine vs Saline	-0.564	0.056	-9.995	0.000	-0.674, -0.453
		TMT vs Saline	-0.491	0.056	-8.775	0.000	-0.601, -0.381
		Animal ID (random intercept)	0.013	0.027			
	Inhibition of 5-HT terminals in BNST (*record in BNST*)	Saline (ref. mean)	0.897	0.072	12.429	0.000	0.756, 1.039
		Urine vs Saline	-0.492	0.092	-5.332	0.000	-0.673, -0.311
		TMT vs Saline	-0.539	0.091	-5.894	0.000	-0.718, -0.360
		Animal ID	0.039	0.067			

		(random intercept)					
	Inhibition of 5-HT terminals in BNST (*record in CeA*)	Saline (ref. mean)	0.744	0.081	9.242	0.000	0.586, 0.902
		Urine vs Saline	-0.434	0.105	-4.148	0.000	-0.640, -0.229
		TMT vs Saline	-0.480	0.105	-4.548	0.000	-0.686, -0.273
		Animal ID (random intercept)	0.047	0.068			
	Activation of 5-HT terminals in CeA (*record in BNST*)	Saline (ref. mean)	1.036	0.065	15.929	0.000	0.908, 1.163
		Urine vs Saline	-0.492	0.083	-5.958	0.000	-0.654, -0.330
		TMT vs Saline	-0.382	0.083	-4.622	0.000	-0.544, -0.220
		Animal ID (random intercept)	0.056	0.058			
	Activation of 5-HT terminals in CeA (*record in CeA*)	Saline (ref. mean)	0.961	0.058	16.437	0.000	0.847, 1.076
		Urine vs Saline	-0.458	0.078	-5.904	0.000	-0.610, -0.306
		TMT vs Saline	-0.562	0.078	-7.252	0.000	-0.714, -0.410
		Animal ID (random intercept)	0.030	0.045			
	Inhibition of 5-HT terminals in CeA (*record in BNST*)	Saline (ref. mean)	0.776	0.066	11.699	0.000	0.646, 0.906
		Urine vs Saline	-0.499	0.078	-6.397	0.000	-0.652, -0.346
		TMT vs Saline	-0.475	0.078	-6.092	0.000	-0.628, -0.322
		Animal ID (random intercept)	0.065	0.078			
	Inhibition of 5-HT terminals in CeA (*record in CeA*)	Saline (ref. mean)	0.934	0.059	15.719	0.000	0.818, 1.050
		Urine vs Saline	-0.504	0.074	-6.858	0.000	-0.649, -0.360
		TMT vs Saline	-0.415	0.074	-5.619	0.000	-0.560, -0.270
		Animal ID (random intercept)	0.073	0.062			
Peak firing rate (Hz)	Activation of 5-HT terminals in BNST (*record in BNST*)	Saline (ref. mean)	1.424	0.059	24.244	0.000	1.308, 1.539
		Urine vs Saline	-0.010	0.082	-0.118	0.906	-0.171, 0.152
		TMT vs Saline	-0.056	0.082	-0.675	0.000	-0.217, 0.106
		Animal ID (random intercept)	0.009	0.035			
		Saline (ref. mean)	0.961	0.066	14.463	0.000	0.831, 1.092

	Activation of 5-HT terminals in BNST (*record in CeA*)	Urine vs Saline	0.074	0.092	0.803	0.422	-0.107, 0.256
		TMT vs Saline	0.074	0.092	0.799	0.424	-0.107, 0.255
		Animal ID (random intercept)	0.027	0.041			
	Inhibition of 5-HT terminals in BNST (*record in BNST*)	Saline (ref. mean)	1.284	0.087	14.719	0.000	1.113, 1.455
		Urine vs Saline	0.148	0.117	1.267	0.205	-0.081, 0.377
		TMT vs Saline	-0.000	0.117	-0.002	0.999	-0.229, 0.229
		Animal ID (random intercept)	0.065	0.064			
	Inhibition of 5-HT terminals in BNST (*record in CeA*)	Saline (ref. mean)	1.071	0.066	16.249	0.000	0.942, 1.200
		Urine vs Saline	-0.040	0.091	-0.443	0.658	-0.220, 0.139
		TMT vs Saline	0.126	0.091	1.379	0.168	-0.053, 0.305
		Animal ID (random intercept)	0.020	0.042			
	Activation of 5-HT terminals in CeA (*record in BNST*)	Saline (ref. mean)	1.018	0.127	8.017	0.000	0.769, 1.267
		Urine vs Saline	0.306	0.179	1.716	0.086	-0.044, 0.656
		TMT vs Saline	0.168	0.179	0.939	0.348	-0.182, 0.518
		Animal ID (random intercept)	0.016	0.075			
	Activation of 5-HT terminals in CeA (*record in CeA*)	Saline (ref. mean)	1.210	0.071	17.093	0.000	1.071, 1.348
		Urine vs Saline	-0.054	0.094	-0.577	0.564	-0.240, 0.131
		TMT vs Saline	-0.038	0.094	-0.398	0.691	-0.223, 0.148
		Animal ID (random intercept)	0.052	0.053			
	Inhibition of 5-HT terminals in CeA (*record in BNST*)	Saline (ref. mean)	1.513	0.129	11.726	0.000	1.260, 1.765
		Urine vs Saline	0.211	0.149	1.415	0.157	-0.081, 0.503
		TMT vs Saline	0.080	0.149	0.538	0.590	-0.212, 0.372
		Animal ID (random intercept)	0.559	0.152			
	Inhibition of 5-HT terminals in CeA (*record in CeA*)	Saline (ref. mean)	1.845	0.120	15.411	0.000	1.611, 2.080
		Urine vs Saline	0.044	0.168	0.264	0.792	-0.285, 0.374
		TMT vs Saline	0.356	0.168	2.114	0.035	0.026, 0.686

		Animal ID (random intercept)	0.036	0.071			
Peak position (s)	Activation of 5-HT terminals in BNST (*record in BNST*)	Saline (ref. mean)	7.661	0.531	14.421	0.000	6.620, 8.702
		Urine vs Saline	-0.575	0.734	-0.783	0.434	-2.014, 0.865
		TMT vs Saline	-0.908	0.734	-1.237	0.216	-2.348, 0.531
		Animal ID (random intercept)	2.318	0.342			
	Activation of 5-HT terminals in BNST (*record in CeA*)	Saline (ref. mean)	6.774	0.509	13.310	0.000	5.776, 7.771
		Urine vs Saline	1.268	0.706	1.795	0.073	-0.116, 2.652
		TMT vs Saline	0.788	0.706	1.116	0.264	-0.596, 2.172
		Animal ID (random intercept)	1.843	0.322			
	Inhibition of 5-HT terminals in BNST (*record in BNST*)	Saline (ref. mean)	7.314	0.779	9.384	0.000	5.786, 8.842
		Urine vs Saline	0.182	1.049	0.174	0.862	-1.874, 2.238
		TMT vs Saline	0.500	1.049	0.476	0.634	-1.556, 2.555
		Animal ID (random intercept)	4.887	0.562			
	Inhibition of 5-HT terminals in BNST (*record in CeA*)	Saline (ref. mean)	7.042	0.616	11.432	0.000	5.835, 8.250
		Urine vs Saline	0.308	0.871	0.354	0.723	-1.399, 2.016
		TMT vs Saline	-0.621	0.871	-0.712	0.476	-2.328, 1.087
		Animal ID (random intercept)	0.000	0.360			
	Activation of 5-HT terminals in CeA (*record in BNST*)	Saline (ref. mean)	6.504	0.811	8.020	0.000	4.914, 8.093
		Urine vs Saline	-1.035	1.114	-0.929	0.353	-3.219, 1.149
		TMT vs Saline	-0.037	1.114	-0.033	0.974	-2.221, 2.147
		Animal ID (random intercept)	2.824	0.536			
	Activation of 5-HT terminals in CeA (*record in CeA*)	Saline (ref. mean)	8.055	0.811	9.929	0.000	6.465, 9.645
		Urine vs Saline	-0.788	1.126	-0.700	0.484	-2.995, 1.420
		TMT vs Saline	-1.565	1.126	-1.389	0.165	-3.772, 0.643
		Animal ID (random intercept)	2.081	0.512			

	Inhibition of 5-HT terminals in CeA (*record in BNST*)	Saline (ref. mean)	6.817	0.578	11.786	0.000	5.683, 7.950
		Urine vs Saline	-0.431	0.789	-0.547	0.585	-1.978, 1.115
		TMT vs Saline	0.218	0.789	0.276	0.782	-1.329, 1.765
		Animal ID (random intercept)	2.445	0.394			
	Inhibition of 5-HT terminals in CeA (*record in CeA*)	Saline (ref. mean)	6.646	0.391	17.016	0.000	5.881, 7.411
		Urine vs Saline	-0.173	0.530	-0.327	0.743	-1.211, 0.865
		TMT vs Saline	0.310	0.530	0.585	0.559	-0.728, 1.348
		Animal ID (random intercept)	2.295	0.273			

Parameter	Group	Fixed & random effects	Estimate (β)	SE	z	p-value	95% CI
Firing rate (FR, Hz)	BNST (*GABAergic neurons*)	Saline (ref. mean)	0.818	0.106	7.692	0.000	0.610, 1.027
		Urine vs Saline	-0.254	0.117	-2.167	0.030	-0.484, -0.024
		TMT vs Saline	-0.145	0.117	-1.234	0.217	-0.374, 0.085
		Animal ID (random intercept)	0.143	0.143			
	CeA (*GABAergic neurons*)	Saline (ref. mean)	1.340	0.399	3.358	0.001	0.558, 2.122
		Urine vs Saline	0.933	0.282	3.307	0.001	0.380, 1.487
		TMT vs Saline	0.636	0.282	2.255	0.024	0.083, 1.190
		Animal ID (random intercept)	8.592	1.159			
	DRN (Seroton*ergic neurons*)	Saline (ref. mean)	0.640	0.106	6.032	0.000	0.432, 0.848
		Urine vs Saline	-0.172	0.133	-1.297	0.195	-0.433, 0.088
		TMT vs Saline	0.006	0.133	0.046	0.963	-0.254, 0.267
		Animal ID (random intercept)	0.046	0.102			
Maintained firing rate (Maint'd FR, Hz)	BNST (*GABAergic neurons*)	Saline (ref. mean)	0.571	0.804	0.710	0.478	-1.005, 2.147
		Urine vs Saline	1.440	1.137	1.266	0.205	-0.789, 3.668
		TMT vs Saline	0.164	1.137	0.144	0.886	-2.065, 2.392
		Animal ID (random intercept)	0.000	0.510			
	CeA (*GABAergic neurons*)	Saline (ref. mean)	396.908	589.802	0.673	0.501	-759.1, 1552.9
		Urine vs Saline	-391.276	738.702	-0.530	0.596	-1839.1, 1056.6
		TMT vs Saline	988.995	738.702	1.339	0.181	-458.8, 2436.8
		Animal ID (random intercept)	2250776.778	559.757			
	DRN (Seroton*ergic neurons*)	Saline (ref. mean)	1.051	0.544	1.933	0.053	-0.015, 2.117
		Urine vs Saline	0.670	0.665	1.008	0.313	-0.633, 1.973
		TMT vs Saline	0.782	0.665	1.177	0.239	-0.521, 2.085
		Animal ID (random intercept)	1.271	0.564			
